# Transplantation of photobiomodulation-preconditioned diabetic stem cells accelerates ischemic wound healing in diabetic rats

**DOI:** 10.1186/s13287-020-01967-2

**Published:** 2020-11-25

**Authors:** Houssein Ahmadi, Abdollah Amini, Fatemeh Fadaei Fathabady, Atarodsadat Mostafavinia, Fatemeh Zare, Roohollah Ebrahimpour-malekshah, Mustafa Neshat Ghalibaf, Matin Abrisham, Fatemehalsadat Rezaei, Richard Albright, Seyed Kamran Ghoreishi, Sufan Chien, Mohammad Bayat

**Affiliations:** 1 Department of Biology and Anatomical Sciences, Shahid Beheshti University of Medical Sciences, Tehran, Iran; 2 Department of Anatomy, Faculty of Medicine, Tehran Medical sciences, Islamic Azad University, Tehran, Iran; 3 University of Kentucky, College of Pharmacy, 789 South Limestone, Lexington, Kentucky 40536 USA; 4 SUMMUS Medical Laser, 1185 W. Main St, Franklin, TN 37064 USA; 5 Department of Statistics, Qom University, Qom, Iran; 6 Price Institute of Surgical Research, University of Louisville, and Noveratech LLC, Louisville, KY USA

**Keywords:** Diabetes mellitus Ischemia, Wound healing, Preconditioning stem cell, photobiomodulation, Adipose-derived mesenchymal stem cells, Tensiometric properties, Methicillin-resistant *Staphylococcus aureus* infection, Wound closure rate, Rats

## Abstract

**Background:**

Diabetic foot ulcer is the most costly and complex challenge for patients with diabetes. We hereby assessed the effectiveness of different preconditioned adipose-derived mesenchymal stem cells (AD-MSCs) and photobiomodulation protocols on treating an infected ischemic wound in type 1 diabetic rats.

**Methods:**

There were five groups of rats: (1) control, (2) control AD-MSCs [diabetic AD-MSCs were transplanted (grafted) into the wound bed], (3) AD-MSC + photobiomodulation in vivo (diabetic AD-MSCs were grafted into the wound, followed by in vivo PBM treatment), (4) AD-MSCs + photobiomodulation in vitro, and (5) AD-MSCs + photobiomodulation in vitro + in vivo.

**Results:**

Diabetic AD-MSCs preconditioned with photobiomodulation had significantly risen cell function compared to diabetic AD-MSC. Groups 3 and 5 had significantly decreased microbial flora correlated to groups 1 and 2 (all, *p* = 0.000). Groups 2, 3, 4, and 5 had significantly improved wound closure rate (0.4, 0.4, 0.4, and 0.8, respectively) compared to group 1 (0.2). Groups 2–5 had significantly increased wound strength compared to group 1 (all *p* = 0.000). In most cases, group 5 had significantly better results than groups 2, 3, and 4.

**Conclusions:**

Preconditioning diabetic AD-MSCs with photobiomodulation in vitro plus photobiomodulation in vivo significantly hastened healing in the diabetic rat model of an ischemic infected delayed healing wound.

## Introduction

Worldwide, diabetes mellitus (DM) is a persistent, challenging metabolic condition for patients, their families, and the community [1]. An estimated 463 million persons suffer from DM globally; this number is anticipated to increase by 25% in 2030 [2]. Approximately 50% of patients with DM are undiagnosed [3]. Diabetic foot ulcer (DFU) is the most costly and complex challenge for patients with DM [4]. DFU impacts 25% of these people at some point in their lives, and over half of these ulcers become infected [4]. Almost 60% of whole limb amputations are performed in people with DM [5]. In most cases, limb amputations are preceded by an infected DFU [6]. *Staphylococcus aureus* is the predominant microbe in infected DFUs [7]. Methicillin-resistant *Staphylococcus aureus* (MRSA) comprises 15–30% of microbial DFUs [7]. Due to overuse of inappropriately prescribed antibiotics, there is an increase in drug-resistant microbes, especially in patients with DFUs [8].

Skin wound healing is an active, natural course of healing that can be separated into the following phases: hemostasis, inflammation, proliferation, and maturation. DM leads to compromised wound repair by disturbances in one or more of the above mentioned phases [9]. Persistent and poorly controlled hyperglycemia, which leads to inflammation, hypoxia, peripheral neuropathy, and ischemia, causes foot deformities and DFU [10, 11]. Compromised injury repair in DM is categorized by decreases in new blood vessel formation, endothelial progenitor cell (EPC) employment, and fibroblast and keratinocyte proliferation and migration [12]. DFUs are considered to be a primary medical challenge [13].

Adipose-derived mesenchymal stem cells (AD-MSCs) could improve DFU repair via boosting re-epithelialization and the creation of granulation tissue, anti-inflammatory and anti-apoptotic effects, and secretion of angiogenic growth factors [14]. Bioactive molecules released by AD-MSC promote new blood vessel formation in an ischemic limb via paracrine actions [15] and improve anti-inflammatory impacts in the injured regions [16]. Despite their potential, barriers should be overcome prior to obtaining the full benefits of AD-MSC. First, restricted transplantation and viability of AD-MSC at the wound place are primary concerns, and substitutions to maximize AD-MSC potential are a major request [17]. Second, autologous cell-based treatments might be restricted by decreasing cellular function related to DM [18]. These documents point out effectiveness of autologous mesenchymal stem cell (MSC) as an alternative treatment in DM might be restricted and some mediations to advance cell action prior application are necessary [17, 19].

Using preconditioning stem cells with photobiomodulation (PBM) is an important strategy to overcome poor engraftment and survival and DM-related impairments in diabetic stem cells. PBM stimulates healing, decreases pain and inflammation [20], and diminishes M1 macrophages in the triggered macrophages [20]. PBM modulates hypoxia-inducible factor (HIF)-1α expression [21], improves regional blood stream, and enhances tissue healing by encouraging angiogenesis [22]. In particular, a combination of PBM plus non-diabetic allograft AD-MSC successfully healed a delayed healing wound in rats with type 2 DM (DM2) [23].

PBM increases the proliferation rate of cultivated cells, as well as MSCs, in vitro without causing cytotoxic effects [24]. Some experiments have shown that preconditioning cells with PBM could be an original non-intrusive tactic for stem cell engraftment, which would improve cell viability and benefit cardiac regenerative therapy and stimulate the paracrine release of MSCs [25].

Recently, in our lab, we have engrafted healthy AD-MSC cells into wounds of diabetic rats [23, 26]. However, in the current probe, we engrafted preconditioned diabetic AD-MSC with PBM into wounds of diabetic rats. We believe the current probe is more closely resembling the clinical situation in which diabetic patients who suffer from DFUs could be treated with their own preconditioned stem cells with PBM. In the existing probe, we initially preconditioned diabetic AD-MSC with PBM in vitro. Next, we assessed different protocols of using AD-MSC and PBM on healing infected ischemic delayed healing wounds in type 1 diabetic rats (DM1). The best protocol of combined administration of PBM and AD-MSC would accelerate the repair course of DFU in diabetic patients.

## Materials and methods

### Animals and study design

Ethical approval of all techniques on animals was confirmed by the IRB of the Shahid Beheshti University of Medical Sciences (File no: IR.SBMU.MSP.REC.1399.105). The male adult Wistar rats were retained in animal rooms with standard conditions: 12 h dark–12 h synthetic light set, and temperature preserved at 22 ± 2 °C. Initially, we introduced DM1 in 10 adult male Wister rats (in vitro phase). The rats were maintained for 30 days. Next, adipose tissue was extracted from the lower abdominal region of each rat. Cells were separated from the adipose tissue in the laboratory and were categorized as AD-MSC. These AD-MSCs were cultured and expanded in vitro in high glucose medium (25 mmol/L) and were considered to be the diabetic AD-MSC. The diabetic AD-MSCs were preconditioned with PBM. In vitro lab tests showed a remarkable escalation in cell viability along with significant decreases in population doubling time (PDT) and apoptosis rate of the laser-treated AD-MSC compared to the diabetic AD-MSC. Because of the elevations in blood glucose levels, and severely decreased body weights in the diabetic rats, we were unable to use these rats for further experimentation. Thus, a second experiment (in vivo phase) was performed on an additional 30 rats. These rats were considered to have DM1 for 21 days prior to infliction of delayed healing wounds in them. At this point, these rats (30 rats) were randomly allotted to five groups (*n* = 6 per group). Group 1 was the control (placebo) rats that received no intervention. In group 2 (control AD-MSC), we grafted (transplanted) the diabetic AD-MSC into the wounds of the rats in this group. Group 3 (AD-MSC + PBM in vivo) received diabetic AD-MSC grafted into the wounds followed by in vivo administration of PBM in the wound area. In the fourth group (AD-MSC + PBM in vitro), diabetic AD-MSCs preconditioned with PBM were transplanted into the wounds. In the fifth group (AD-MSC + PBM in vitro+ in vivo), diabetic AD-MSCs preconditioned with PBM were transplanted into wounds, and each wound was treated with PBM in vivo. Wound closure rate, microbial examination and colony-forming unit (CFU) counts, wound strength, and stereological tests were assessed. Each of the above mentioned examination was performed in 6 rats. Days 4, 8, 12, and 16 were supposed to be the inflammatory, proliferation, early, and late remodeling phases of the skin wound healing process. Rats were euthanized on day 16, and tensiometerical and histological samples were extracted.

### Preparation of diabetic AD-MSC and in vitro culture

We administered an intraperitoneal (IP) injection of streptozotocin (STZ, 40 mg/kg) to each of the 10 adult male Wistar rats for induction of DM1. DM1 was verified when the rats had blood glucose levels higher than 250 mg/dl [27]. All rats were maintained for 30 days to confirm the establishment of the DM1 model [21]. These rats had severe declines in body weight and adipose tissue. Too little adipose tissue was extracted from the lower abdominal region and inguinal pad fat. AD-MSCs were extracted from the adipose tissue by standard protocol, and the cells were cultured in an elevated glucose concentration (25 mmol/l or 450 mg/dl). Flow cytometry technique was utilized to characterize the AD-MSC in terms of MSC cluster of differentiation markers (CD) (CD11b, CD45, CD44H, and CD105) as previously reported [23].

### One-time diabetic AD-MSC injection and one-time transplantation of preconditioned diabetic AD-MSC

**Fig. 1:**
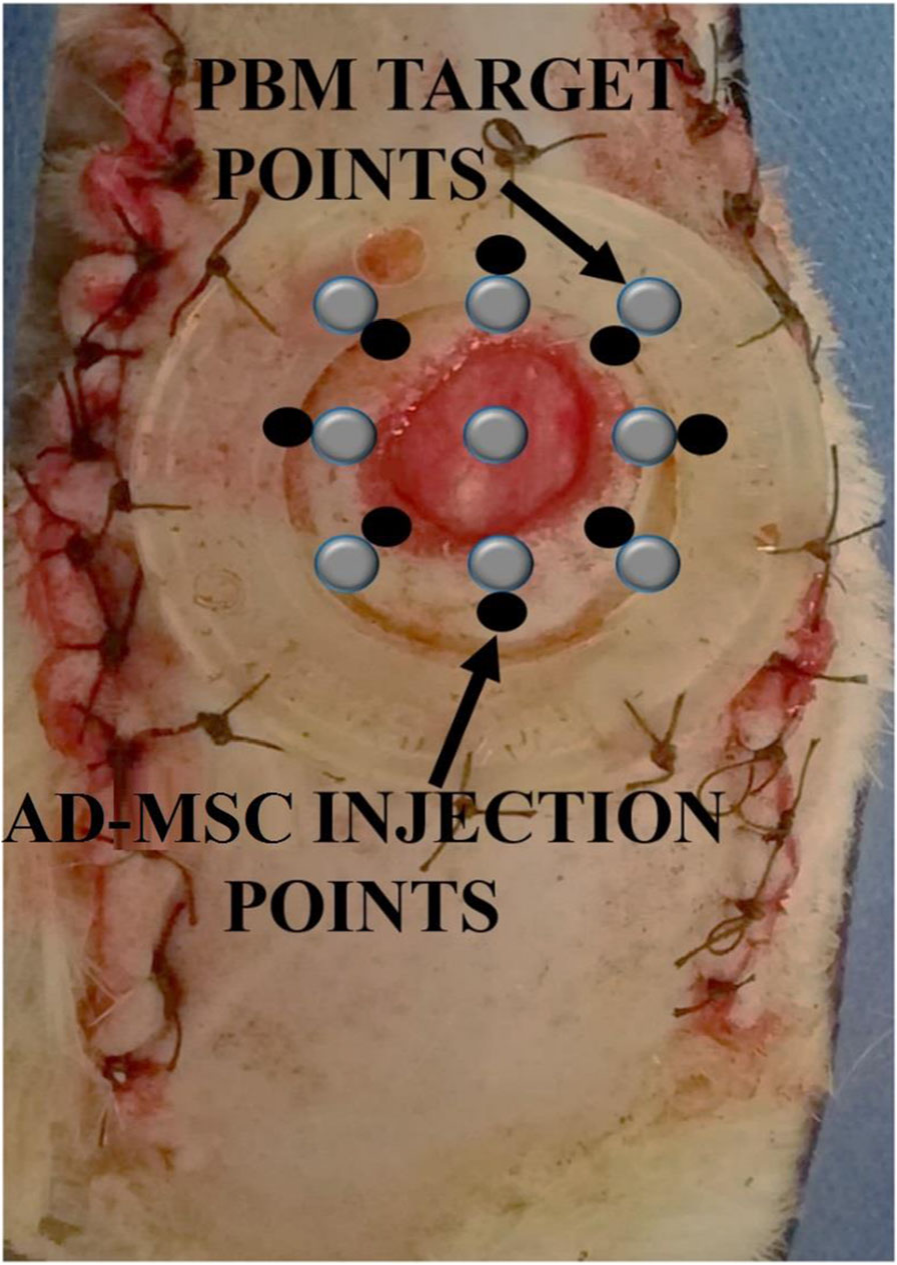
A photo of the wound, photobiomodulation (PBM) target points, and adipose-derived mesenchymal stem cell- (AD-MSC) injection points

At 24 h after surgery, 1 × 10^6^ passage-4 diabetic AD-MSCs in 300 μl PBS were injected intradermally into eight sites around each wound (Fig. 1). Diabetic AD-MSCs were injected into the wounds of rats in group 2, and preconditioned diabetic AD-MSCs were injected into wounds of rats from groups 4 and 5 [23].

### Preconditioning of diabetic AD-MSC with PBM in vitro

1 × 10^4^ passage-4 AD-MSCs were seeded in each well of a 24-well plate for each of the three groups: healthy control AD-MSC, diabetic control AD-MSC, and experimental diabetic AD-MSC. Here, red laser alone plus infrared laser alone at two energy densities were used to irradiate the AD-MSC every other day for three sessions according to our previously published protocol [28]. Table 1 lists the in vitro PBM parameters. At 24 h after the last PBM administration, we used MTT, PDT, and apoptosis rate tests to determine the best PBM protocol for preconditioning the diabetic AD-MSC.

**Table 1:** Specifications of in vitro and in vivo photobiomodulation parameters

**Specifications of in vitro photobiomodulation**							
**Laser type**	**Wavelength (nm)**	**Power (W)**	**Duration of each administration (s)**	**Energy density (J/cm**^**2**^**)**	**Laser beam diameter (cm)**	**Laser beam area (cm**^**2**^**)**	**Power density (W/cm**^**2**^**)**
**Red**	630	0.05	46, 92	1.2,2.4	1.56	1.91	0.0261
**Near infrared**	810	0.05	46, 92	1.2,2.4	1.56	1.91	0.0261
**Company**			NILTVIR202 Noura Instruments, Tehran, Iran				
**Specifications of in vivo photobiomodulation**							
**Parameters**			**Dose and unit**				
**Peak power output**			75 W				
**Average power**			0.001 W				
**Power density**			0.001 W/cm^2^				
**Wavelength**			890 nm				
**Wavelength range of the device**			890±10 nm				
**Pulse frequency**			80 Hz				
**Spot size**			1 cm^2^				
**Diameter**			1.12 cm				
**Pulsed duration**			180 ns				
**Duration of exposure for each point**			200 s				
**Energy density**			0.2 J/cm^2^				
**Number of laser shootings in each session**			9				
**Energy densities for one session and for the total sessions**			1.8 and 25.2 J/cm^2^				
**PBM radiation scheduling**			Immediately after surgery, 6 days per week, for 16 consecutive days				
**Probe**			L07				
**Company**			MUSTANG 2000, Technica Co., Russia				

### MTT test

We used the MTT test to count the numbers of viable cells, and the AD-MSCs were prepared for the MTT test as previously explained [29].

### PDT test

$$ \mathrm{PDT}=T\times \lg 2/\left(\mathrm{lgNt}\hbox{-} \mathrm{lgN}0\right) $$

where *T* = AD-MSC culture time, N0 = initial AD-MSC number, and Nt = number of harvested AD-MSCs [29].

### Acridine orange (AO)/ethidium bromide (EB) staining

We added 200 μl dual fluorescent staining solution that contained 100 μg/ml AO + 100 μg/ml EB (Sigma Aldrich, USA) to each well of the AD-MSC culture plate. Morphology and percentages of live and apoptotic AD-MSCs in five fields were assessed by a fluorescent microscope (Nikon, C-SHG, Japan) and recorded [29].

### Surgery

The 30 diabetic rats underwent surgery as described previously [23]. At first, under general anesthesia and sterile conditions, a bipedicle skin flap (10 × 3.5 cm) was created on the dorsal region of each rat. Next, a 12-mm full-thickness circular excision was generated in the middle of each flap and a silicone made ring frame was fixed around each wound to counteract skin muscle contraction (Fig. 1).

### Gross examinations

The rats’ weights and blood sugar levels throughout the project were documented.

### Inoculation of MRSA into the wounds and microbiological examination

The rats received injections of MRSA according to a previously published protocol [23]. Briefly, each wound was covered with a topical application of a 100-μl aliquot that contained 2 × 10^7^ MRSA (ATCC 25923) just after surgery. Microbiological samples were obtained for routine microbiological analyses on days 8 and 16, and the numbers of probable bacterial colonies in the wound from each rat were reported as the CFU [23].

### In vivo PBM

The wounds of the rats in groups 3 and 5 were subjected to PBM in vivo (Fig. 1). Table 1 lists the complete specifications of the in vivo PBM protocol.

### Wound closure rate

We photographed the wounds on days 0, 4, 8, 12, and 16 and measured the wound surface area by ImageJ software (NIH, USA). The wound closure rate was calculated as follows [26].

$$ \left[\left(\mathrm{Surfacearea}\ \mathrm{at}\ \mathrm{day}\ 0-\mathrm{surfacearea}\ \mathrm{at}\ \mathrm{day}\ X\right)/\mathrm{surfacearea}\ \mathrm{at}\ \mathrm{day}\ 0\right]\times 100\%. $$

### Tensiometric examination

One 5 × 50 mm typical sample from each wound of all the rats was extracted on day 16 and subjected to the deformation rate (0.166 m/s) of a material testing machine. We calculated the bending stiffness (MPa) and stress high load (N/cm^2^) of the samples [23].

### Histological and stereological analyses

Neutrophils, macrophages and fibroblasts count, and vascular lengths measurement were examined in 10 randomly selected slides of each rat. Hematoxylin and eosin (H&E)-stained slides were assessed according to the physical dissector method at magnification of × 400 under a light microscope. Special criteria based on two previously published papers [30, 31] were utilized for the abovementioned cell selection, specifically endothelial cells. Collagen fibers were examined semi-descriptively in Mallory’s trichrome staining slides [23].

### Calculation of the cell numbers

$$ \mathrm{Nv}=\Sigma Q/\left(h\times a/f\times \Sigma \mathrm{p}\right) $$

where Nv is the numerical density, Σ*Q* number of nuclei, *h* height of the dissector and *a*/*f* was all counting frame (field) area in each rat.

*N* (total of cells in each rat) = Nv × *V*

where Nv is the numerical density and *V* the final total volume.

$$ \mathrm{Estimation}\ \mathrm{of}\ \mathrm{vascular}\ \mathrm{length}\ \mathrm{a}\mathrm{s}\ \mathrm{a}\ \mathrm{biomarker}\ \mathrm{for}\ \mathrm{angiogenesis}=2\Sigma Q/\left(\Sigma P\times a/f\right) $$

where 2Σ*Q* (total number of the vessels counted per rat)/ΣP (number of counting frames in all fields (*a*/*f*)) [23].

### Statistical analysis

Data are shown as mean ± standard deviation (SD). The comparison of body weights and blood sugar values was performed by the *t* test. We used one-way analysis of variance (ANOVA), repeated measurement analysis, and least significant difference (LSD) tests for statistical analyses of microbial, wound closure, tensiometerical, and stereological examinations. A logistic regression model fitted to the data was used to estimate the ulcer closure rate (the number of wounds closed/total samples) in each group on day 16. A *p* value of < 0.05 was considered significant.

## Results

### Marker expressions

Flow cytometry analysis showed that the diabetic AD-MSC cells slightly expressed hematopoietic CD markers CDs11b (0.33%) and CD45 (0.8%) and completely expressed mesenchymal CDs 44H and 105 (100%). The graphs are shown in Additional file 1 (Fig. 7).

### In vitro assay results

**Fig. 2:**
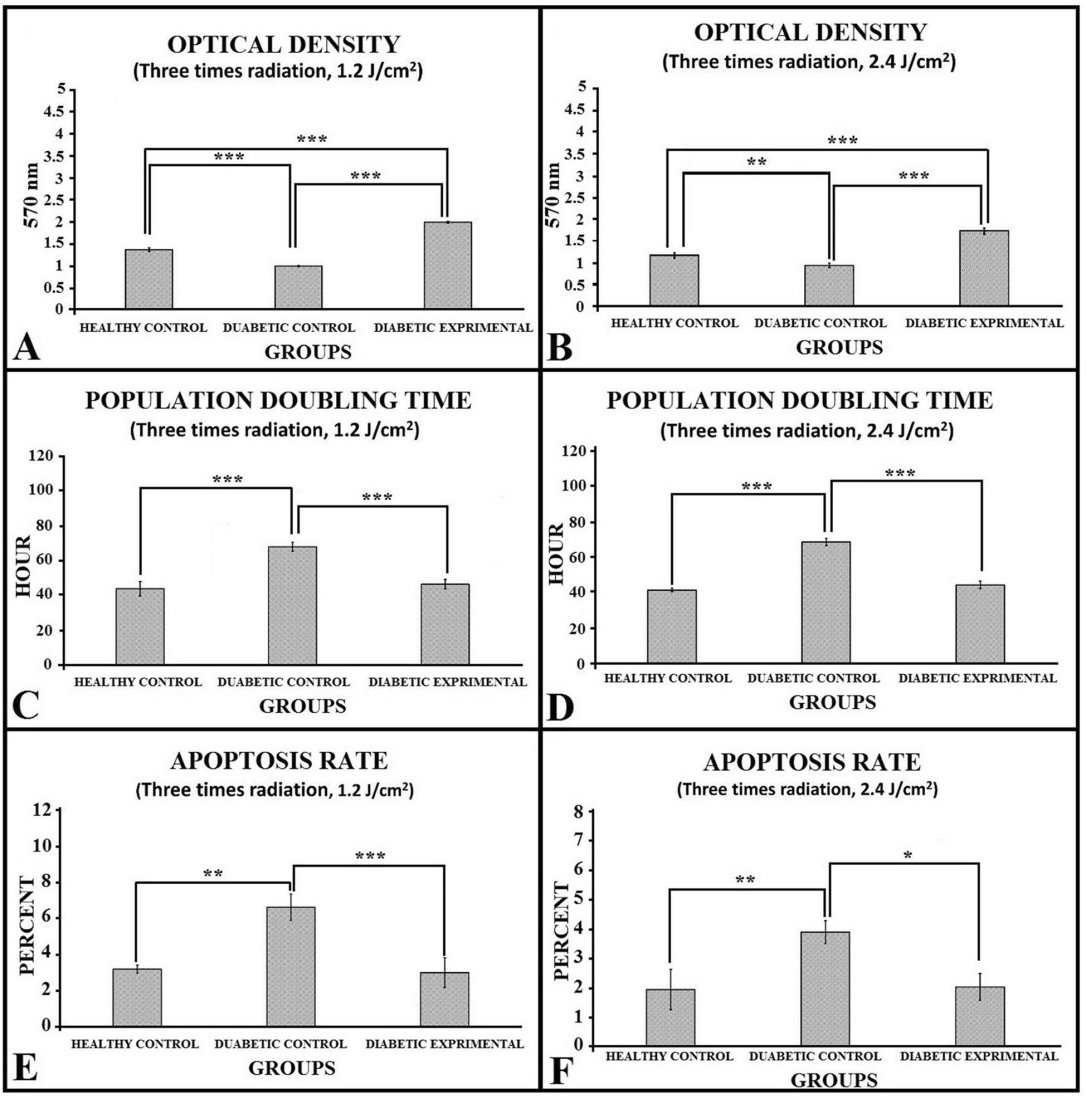
Mean ± SD of the MTT test (**a**, **b**), population doubling time (**c**, **d**), and apoptosis rate results (**e**, **f**) of AD-MSC of the studied groups compared by analysis of variance (ANOVA) and least significant difference (LSD) tests. *p < 0.05; **p < 0.01; ***p < 0.001

Figure 2 shows the in vitro assay results. All *p* values were derived from the LSD test. Briefly, administration of 1.2 J/cm^2^ PBM significantly increased cell survival and significantly decreased PDT and the apoptosis rate in the experimental diabetic AD-MSC group compared with the healthy control AD-MSC and diabetic control AD-MSC groups. Therefore, we selected 1.2 J/cm^2^ PBM for preconditioning the AD-MSC.

### In vivo results

#### Gross observations

All of the rats had significant elevations in blood glucose levels and reductions in body weight after the STZ injection. Details are shown in Table 2 in Additional file 1.

### MRSA findings

**Fig. 3:**
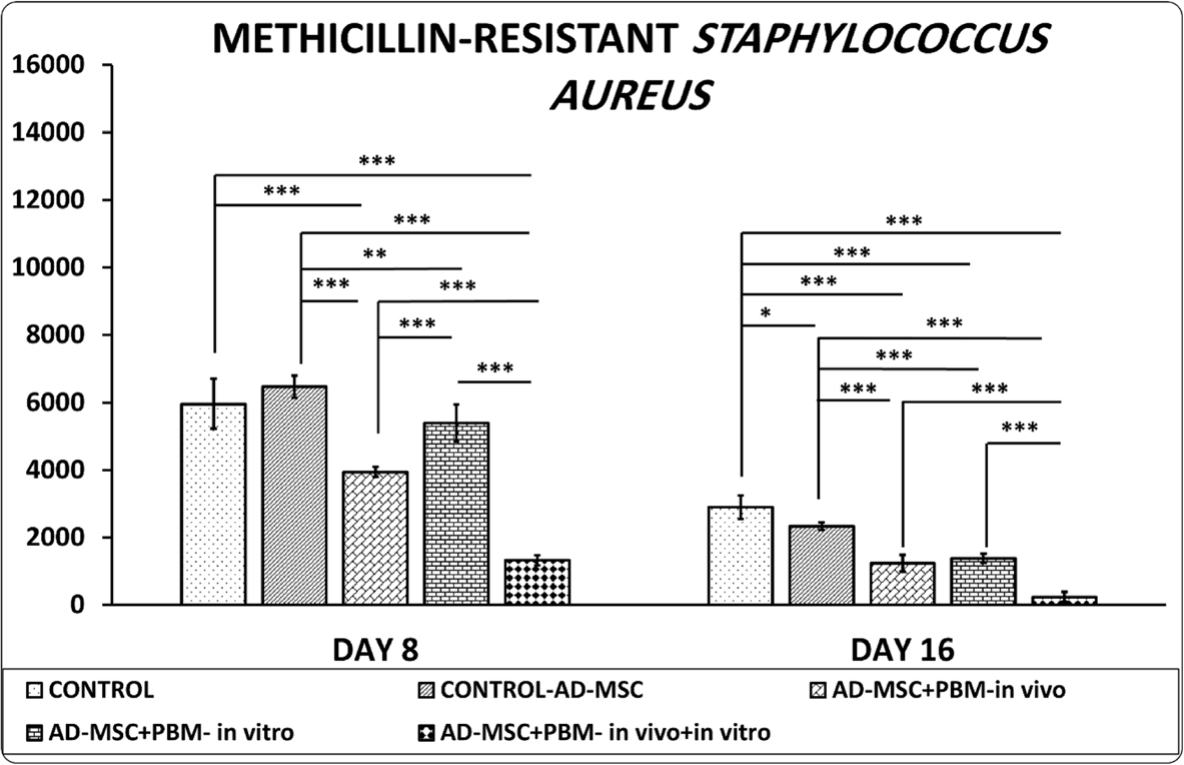
Comparison of colony-forming units of methicillin-resistant *Staphylococcus aureus*-infected wounds in the studied groups by the LSD test. *p< 0.05; **p< 0.01; ***p< 0.001

Figure 3 shows the CFU results in the infected wounds of the study groups. Briefly, all groups with PBM therapy significantly diminished CFUs in the wounds compared (correlated) to the control groups (all, *p* = 0.000). However, group 5 had statistically more effective results than the other groups (all, *p* = 0.000). Treatment with only AD-MSC was not effective.

### Day 8

Groups 5 and 3 significantly decreased CFU in the wounds correlated to groups 1, 2, and 4 (all, *p* = 0.000) (Fig. 3).

### Day 16

Groups 5, 4, and 3 significantly decreased the CFU in the wounds correlated to groups 1 and 2 (all, *p* = 0.000).

### Outcome of wound closure rate examination

**Fig. 4:**
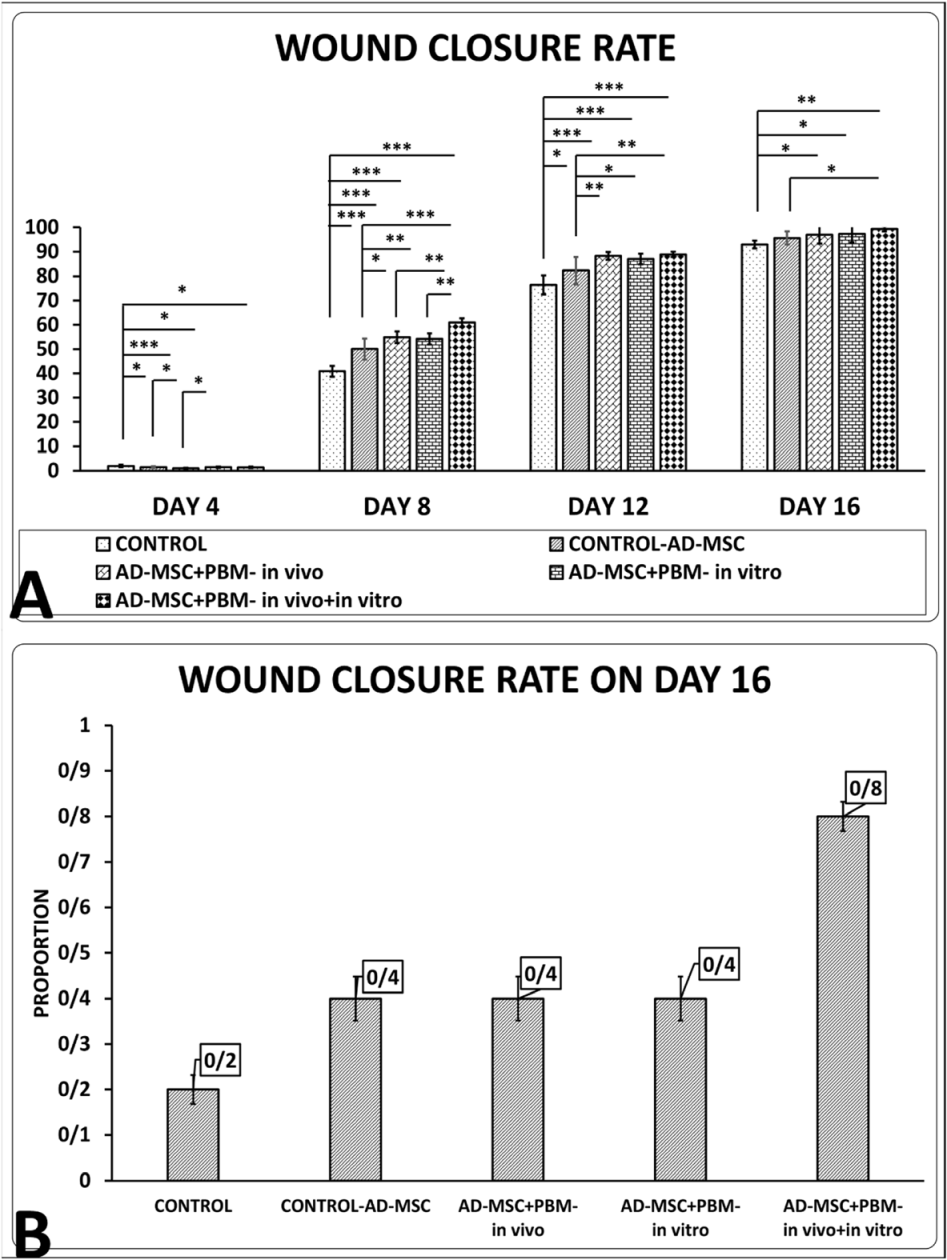
**a** Comparison of wound closure rate from all groups according to the LSD test. The corresponding estimated proportions of the logistic regression model to the data in each group are shown in **b**. *p< 0.05; **p< 0.01; ***p< 0.001

All findings are shown in Fig. 4. In days 8, 12, and 16, the results of groups 3, 4, and 5 were statistically better than those of group 1.

### Day 4 wound closure rate

Group 3 had a significantly decreased wound closure rate correlated to groups 1 (p = 0.000), 2 (*p* = 0.016), and 4 (*p* = 0.033).

### Day 8 wound closure rate

Groups 5 (*p* = 0.000), 3 (*p* = 0.008), and 4 (*p* = 0.02) had significantly increased wound closure rates correlated to groups 1 and 2. Group 5 was significantly better than groups 3 (*p* = 0.002) and 4 (*p* = 0.001).

### Day 12 wound closure rate

Groups 5 (*p* = 0.003), 3, and 4 (*p* = 0.031) had significantly increased wound closure rates correlated to group 2.

### Day 16 wound closure rate

Group 5 was significantly better than group 2 (*p* = 0.033).

### Logistic regression analysis at day 16

Figure 4, panel b, shows the corresponding estimated wound closure rate in each group by logistic regression analysis. The highest wound closure rate was observed in group 5 (0.8). The wound closure rates for groups 2, 3, and 4 were 0.4, and for group 1, it was 0.2.

### Tensiometrical findings

Groups 2–5 had significantly increased bending stiffness and stress high load in the wounds correlated to group 1 (all, *p* = 0.000).

### Bending stiffness and stress high load

**Fig. 5:**
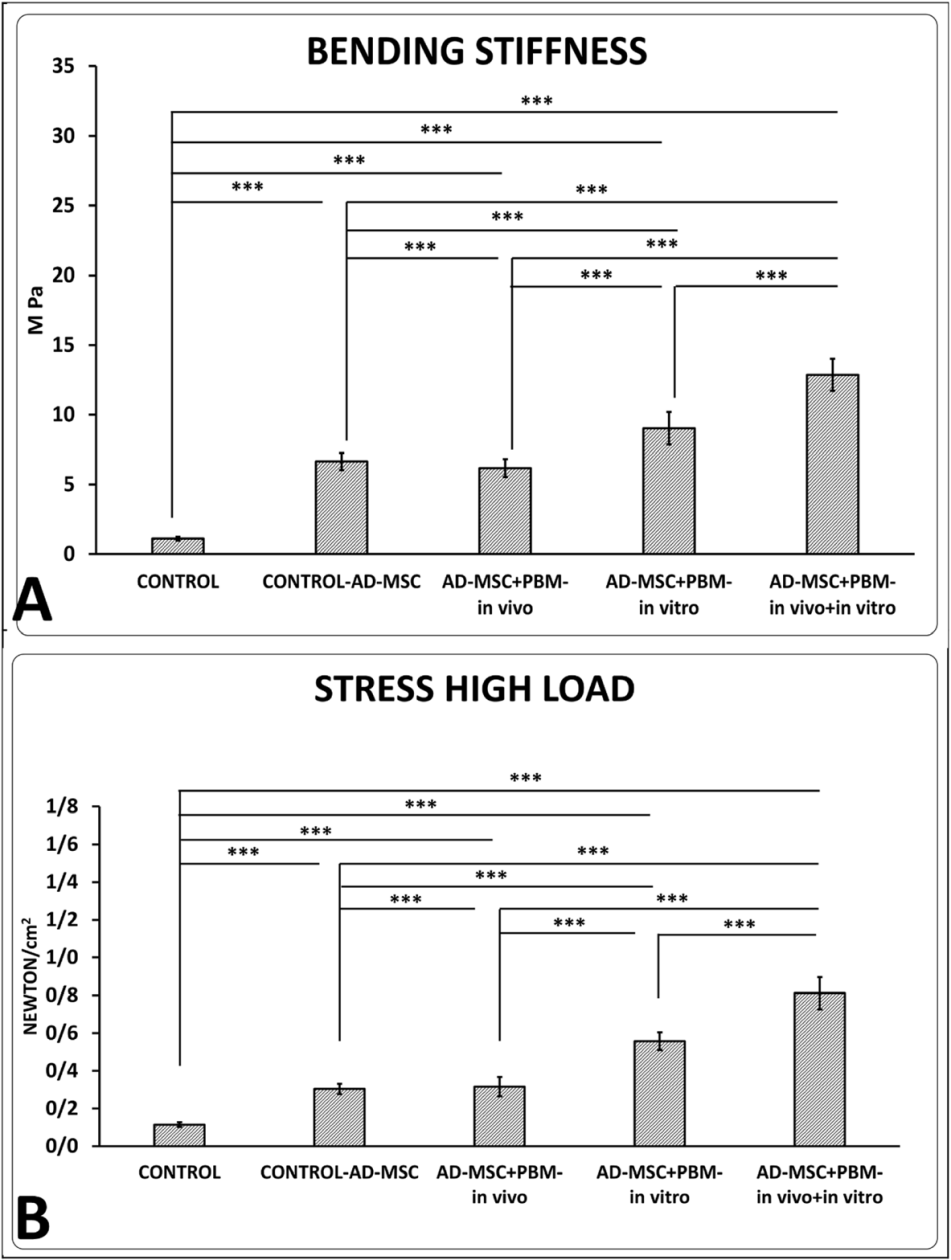
Comparison of bending stiffness (**a**) and stress high load (**b**), of the wounds in the experimental groups according to the LSD test. ***p < 0.001

Groups 5 and 4 had significantly better bending stiffness and stress high load than groups 3 and 2 (all, *p* = 0.000). The results of group 5 were significantly better than those of group 4 (*p* = 0.000) (Fig. 5).

### Outcomes of histological analysis and finding of H&E staining

**Fig. 6:**
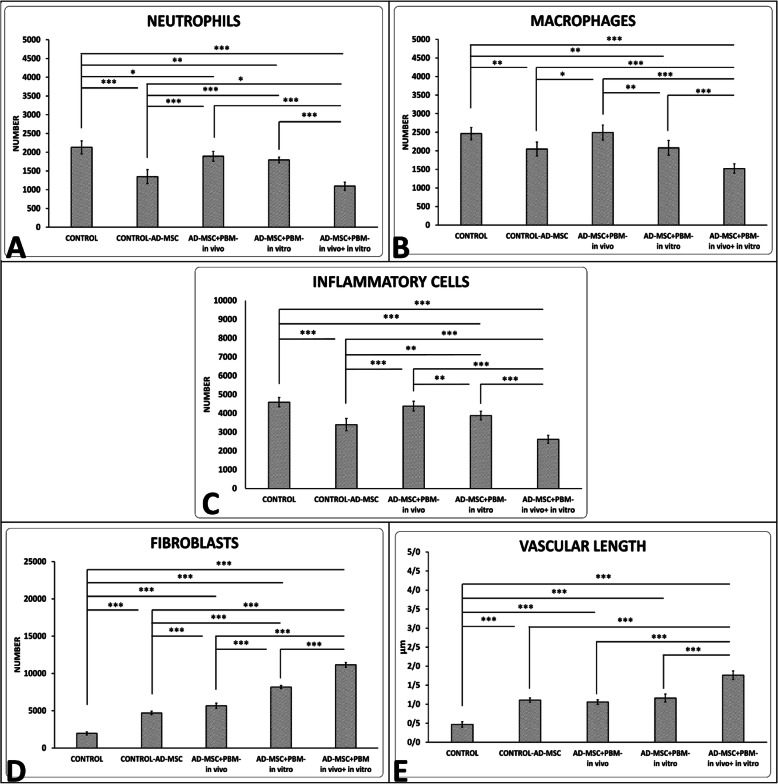
Comparison of numbers of neutrophils (**a**), macrophages (**b**), inflammatory cells (**c**), fibroblasts (**d**), and vascular lengths (**e**) of the wounds from the five study groups by the LSD test. *p < 0.05; **p < 0.01; ***p < 0.001

Photos of histological slides are shown in Additional file 1 (Fig. 8). We found that groups 5 and 2 had significantly modulated inflammatory cells correlated to group 1 (both, *p* = 0.000). Groups 2, 3, 4, and 5 had significantly increased fibroblast counts and vascular length correlated to group 1. The results of group 5 were statistically better than other groups. All results are shown in Fig. 6.

### Finding of Mallory’s trichrome staining

Collagen fiber orientation in the control group was mostly mixed. In most cases, they were horizontal in the treatment groups. Collagen fibers in the control group were mostly thin and loose, whereas in groups 2–5, they were thicker and more compact than in the control group. Mallory’s trichrome staining in the study groups is shown in Additional file 1 (Fig. 9).

## Discussion

Under normal circumstances, the inflammatory phase of the wound repair is well-coordinated, enduring just within days, and the phases of wound repair advance routinely. But, in DM, the inflammatory phase is prolonged and the entirety of the wounded skin is not reestablished, which results in delayed healing or ulcer formation [32]. Patients with DFU should undergo numerous types of treatments for prolonged periods of time, which are costly, and result in a tremendous financial burden for community and insurance organizations [33].

Scientists have focused on innovative tactics to treat delayed healing wounds [34]. MSC cell-based methods have been proposed as potential treatments for delayed healing wounds. Both in vitro and in vivo animal experiments have shown that AD-MSCs differentiate into different types of skin cells and release bioactive molecules that contribute to wound repair in a paracrine way [35]. Autologous MSCs including AD-MSC have been applied in most of medical and animal probes [34]. AD-MSC could escalate the viability of the adipose graft by regulating the inflammatory and oxidative stress responses [36]. The predominant use of autologous MSCs is legitimatized by easier isolation procedures, security, and lack of ethical struggle [37]. So in the current study, we selected an approach toward using autologous stem cell for treating diabetic wounds in future studies.

Despite the benefits, there are numerous problems that preclude stem cell use as a treatment of the wound skin. (1) Elevated blood glucose levels in DM result in the aggregation of advanced glycosylation end products (AGEs). These AGEs suppress proliferation and cause apoptosis of AD-MSC, suppress differentiation of AD-MSC into endothelial cells, and prevent the production of collagen protein, all of which contribute directly to delayed wound healing [38]. (2) Despite the increased use of MSCs in clinical trials, the therapeutic benefits remain insignificant [39] and are partially attributed to the natural restricted illness-adjusting capability of MSCs [39]. On the other hand, tissue damage and the curative body reactions cause secretion of endogenous hazard signals [40, 41], which change the immune micromilieu [42]. Hazard signals adversely affect healing in numerous damaged organs [43]. Therefore, the discovery of novel approaches that increase the capabilities of the MSCs is a dynamic field of biological investigation that has medical significance [39]. MSCs are one of the most studied choices for stem cell treatment [44]. (3) Decreases in the amount of stem cells in some experimental models of skin wound have been reported. Wu et al. observed a rapid decrease in stem cell survival 14 days after induction of a wound [45]. Muhammad et al. reported decreased survival of transplanted AD-MSC in an animal model of a burn injury [28]. However, despite the poor function of transplanted AD-MSC at the wound site, the use of certain special pretreatment agents not only provided a good biological environment that enhances survival of the AD-MSC, but also promotes proliferation, differentiation, and paracrine abilities of these AD-MSCs [46]. PBM has been shown to increase the proliferation ratio of cultivated AD-MSC [29], and it is an effective tactic for preconditioning AD-MSC in a culture system prior to implantation [47]. PBM therapies are non-intrusive, cost-effective modalities, and wise choice instruments for wound treatment. PBM-based clinical investigations could indicate novel areas for the use of PBM and MSCs as treatments.

In the current study, we observed that preconditioning diabetic AD-MSC with PBM significantly increased the survival of diabetic AD-MSC and significantly diminished PDT and the apoptosis rate of this group compared with the diabetic control AD-MSC group in vitro (Fig. 2). Our in vivo analysis showed that in terms of tensiometric and stereological evaluating methods, groups 2–5 had significantly improved tensiometric and stereological parameters of the wounds compared to the control group (all, *p* = 0.000). The AD-MSC + PBM in vitro + in vivo group was significantly better than the other groups in terms of these tensiometric and stereological parameters (all, *p* = 0.000). The results indicated that preconditioning diabetic AD-MSC with PBM prior to transplantation of these AD-MSCs into the wound bed and subsequent treatment of the wounds with in vivo PBM could overcome the barriers to wound healing in a delayed healing wound. This group had the best outcomes from the different protocols of combined AD-MSC and PBM in the current study. These results were partly in line with those reported by Liu and Zhang and Liao et al. In a review article, Liu and Zhang concluded that conditioning with PBM provoked proliferation, differentiation, and paracrine release of MSCs for cardiac renewal therapy [25]. Liao et al. examined the impact of preconditioned human (h) AD-MSC with PBM (650 nm, 2–8 J/cm^2^) in an in vitro aging skin mouse model. They reported that preconditioned hAD-MSC with PBM markedly improved damaged skin. Liao et al. concluded that PBM was a persuasive bioenhancer of hAD-MSC and could stimulate the beneficial function of AD-MSC for treatment [27].

Here, we showed that although transplantation of diabetic AD-MSC accelerated the healing process of an ischemic, delayed healing, MRSA-infected wound, however, preconditioning of diabetic AD-MSC with PBM provided synergistic benefits. Accordingly, Muhammad et al. have shown that transplantation of AD-MSC accelerated the healing process of an acid burn wound. Preconditioning of the AD-MSC provided additional benefits [28].

From a clinical perspective, the key challenge will be translating insights from hair follicle (HF) biology into treatment of disorders such as wound healing and tissue regeneration, as well as de novo induction of HFs in adult human skin. Since the HF and its surrounding mesenchyme are potent sources of multipotent stem cell populations, this raised the hope for the application of stem cells within adult human HFs in regenerative medicine [48]. Recent studies have shown that stem cells residing in HFs are able to contribute in re-epithelialization and wound closure in vivo [48, 49]. We did not directly study the role of stem cells from wound adjacent HF in the wound closure. Inhibiting the wound closure of skin muscle through applying a ring frame around the wound iterated prominent regenerative role of keratinocytes, and adjacent HF stem cells. Accordingly, wound closure in groups 3–5 was significantly better than groups 1 and 2. At the same time, the results of group 5 were significantly better than other groups. It means that in terms of wound closure, the combined application of preconditioned diabetic AD-MSC with PBM in vitro plus PBM therapy in vivo has a significantly superior effect compared to the other treatments.

In the present study, we observed that an anti-inflammatory agent and a mitogenic agent, PBM [20], exhibited a synergistic effect on diabetic AD-MSC induced wound healing in vivo [35]. Diabetic AD-MSC alone significantly induced an anti-inflammatory effect when compared with groups 1, 3, and 4. Our results also demonstrated a significantly decreased inflammatory response after treatment with PBM preconditioned diabetic AD-MSC plus PBM in vivo (group 5) compared to treatment with AD-MSC alone. The improved proliferative activity (increased fibroblast counts) in the wound bed of the diabetic AD-MSC was specifically increased after preconditioning with PBM (Fig. 6).

In terms of bactericidal impact, treatment with only diabetic AD-MSC was not effective (Fig. 3). We observed that the combination of AD-MSC + PBM in vitro was significantly superior to treatment with only AD-MSC with regard to wound closure rate at day 8 and stress high load, bending stiffness, fibroblast counts, and bactericidal effects. These findings have implied that although the appropriate PBM protocol administered in vitro could facilitate the repair capabilities of transplanted diabetic AD-MSC in a DM1 model of an MRSA-infected wound and accelerate the wound healing process, the combined AD-MSC + PBM in vitro + in vivo (group 5) was significantly superior. In total, these results suggested that preconditioning with PBM, which is a cost-effective [50], anti-inflammatory, and mitogen agent [20], could be a powerful supplement for diabetic AD-MSC-based therapy in treating DFUs.

The promising results of the current study of preconditioning diabetic AD-MSC with PBM, which significantly increases both viability and function of AD-MSC in vitro and in vivo, have encouraged us to suggest additional in vitro and in vivo studies with animal models of wound healing and patients who suffer from DM. Hopefully, the final outcomes of these studies will enable the use of PBM plus diabetic (autologous) AD-MSC protocols to attain beneficial responses for DFU repair in patients with DM.

While diabetic rats lost significantly their weight after STZ injection in in vivo part, we could not hold them for more than 21 days before surgery. This is considered as a limitation of our work. As we mentioned in the study design, wound closure rate, microbial examination and CFU counts, wound strength, and stereological tests were performed in each wound of rats, so we decreased the number of rats four folds in the current study and save the life of many animals.

Previously, co-treatment of AD-MSC with decellularized extracellular matrices (ECM) [49], and stromal vascular fraction (SVF) of nanofat [50] have been examined on several complications such as DFUs and scars. The data recommended the safety and efficiency of allogenic AD-MSC and ECM engraftments with no prominent complications [49]. There was also an encouraging relationship between SVF yield and medical effects in the nanofat treatment of scars [50]. In the lack of a uniform technique, renewing allogenic treatments by decellularized ECM would be tough, if not just untrustworthy [49]. Applying nanofat for curing scars requires more attention in terms of the medical ethic issue as well. Platelet-rich plasma (PRP) is a distillate of platelets and cytokines attained by the centrifugation of venous. In vivo application of PRP as a harmless and persuasive procedure reviewed by having an encouraging influence on tissue repair. This review article has some limits that might pose danger of likely prejudices. De Angelis et al. evaluated bio-functionality of a PRP-hyaluronic acid (HA) composed scaffold in comparison to traditional dressings (HA alone, control group) in an in vitro and in vivo experiment in patients with DFUs and vascular ulcers. One month and 80 days later, De Angelis et al. observed significantly better results in the patients with combined PRP + HA therapy than the control group. De Angelis et al. concluded that PRP + HA regime showed stronger renewing potential in terms of keratinocyte proliferation and dermal restoration compared with HA alone [51]. Skin grafts have been used to restore acute and chronic wound insufficiencies with dissimilar etiologies. Nevertheless, the obtainability of adequate well skin, and disfigured donor site morbidity should be issued and have to be deliberated when choosing for skin transplantation.

Recently, researchers and surgeons have worked together to develop various bioengineered and artificial substitutes to encourage tissue renewal in cutaneous wounds [52]. Double layer dermal substitute (DS) contains a 3-dimensional collagen structure and a superficial silicon layer which are positioned within the wound supply to stimulate tissue restoration in cutaneous wounds. Accordingly, De Angelis et al. compared two kinds of DSs (Nevelia®, an innovative collagen DS, and Integra) in patients with post-traumatic injury wounds. De Angelis et al. observed that at long-term follow-up, Nevelia had a better clinical outcome, more angiogenesis, and tissue regeneration compared with Integra [53]. In another investigation, De Angelis et al. reviewed the surgical results of 20 patients, who experienced the application of Dermal Regeneration Template (DRT) for scalp reconstruction for minor defects. During 3 weeks, De Angelis et al. observed the complete healing of the wound bed by secondary intention with suitable cosmetic outcomes and firm scars [54]. Currently, no consistent technique for the extraction of adipose tissue exists. Consequently, Gentile et al. stated that autologous therapies using adipose-derived stromal vascular fraction (AD-SVF) and AD-MSCs warrant careful preparation for being harvested from adipose tissue. Gentile et al. suggested some quantitative and qualitative standards for extracting adipose tissue. Gentile et al. found that the discovery of new critical quality attributes (CQAs) of AD-MSCs evolves with respect to purity and potency. Adjustments to these benchmark standards will hopefully isolate AD-MSCs of various CQAs with greater reproducibility, quality, and safety. However, confirmatory studies undoubtedly need to be completed [55]. In the last study, De Angelis et al. treated 35 patients who suffered from chronic vascular ulcers with Nevelia® followed by autologous dermal-epidermal graft (DEG). De Angelis et al. found that the medical findings of the Nevelia group were better than the control group. Correlated histological and medical findings showed a better skin regeneration with a new formed tissue architecture analog to normal physiology of the skin in the Nevelia group [56].

It is necessary to improve and expand this field in a way that stem cell therapy and biotechnology can be applied cooperatively by adding different in vitro and in vivo applications of AD-MSCs, SVFs, and autologous growth factors like to PRP in tissue regeneration. For this reason, in spite of animal studies, it is necessary to report the application of SVFs and AD-MSCs in improving wound healing when utilized alone or in combination with HA, PRP, and fat graft, in humans. These experiments should focus on improving wound healing in humans as in vitro and pre-clinical conducted studies.

MSC-based therapeutics offers a novel approach toward chronic non-healing wounds. Stem cells exert their effects primarily through cytokine signaling. Combined secretion of growth factors and cytokines has been shown to promote wound repair. This combination of growth factors and cytokines successfully reduces inflammation and promotes angiogenesis, fibroblast migration, and collagen production as we observed in the current work. This environment contributes to healing and improves underlying pathologies, decreasing the recurrence of wounds and ulcers [51]. Stem cell proliferation and signaling play crucial roles in every phase of the wound healing process. Chronic wounds are often associated with impaired stem cell function. Although widespread adoption of stem therapy has been complicated by the costs and complications associated with large-scale production of cell products, cell-based therapy for non-healing wounds is a field with great potential. Increased population ages and the number of diabetic patients have increased the costs of chronic wound care. Improving new treatment strategies would help the patient to cope with this situation [51, 52].

While many of current treatment protocols for the management of wounds and ulcers are expensive and invasive [53], PBM techniques, in the shape of multipurpose light devices, are a noninvasive, economical, and attractive tool for wound management [25].

Many reports showed AD-MSC-based cell therapy products have optimal efficacy and efficiency in some clinical indications in both autologous and allogeneic purposes. Hence, they are being considered as potential tools for replacing, repairing, and regenerating dead or damaged cells. In this section, the therapeutic advancement of AD-MSCs in comparison to bone marrow (BM) MSC and some old reconstructive surgery methods were analyzed. AD-MSCs from adipose tissue and buccal fat pad, as easily harvestable and accessible sources, have gained interest to be used for some reconstructive surgeries and bone regeneration in the maxillofacial region and other parts of the body [54]. However, bone marrow mesenchymal stem cells (BMMSC) harvesting is a highly invasive and painful procedure implying general anesthesia and many days for hospital care. BMMSCs constitute a rare population, with only 0.002% of the total stromal cell population, and their isolation depends on the patient status and the volume of aspirates. AD-MSCs are currently isolated from the subcutaneous adipose tissue, which allows the rapid acquirement of large numbers of highly active cells. The SVF harbor nearly 2% of AD-MSCs which is one of the greatest amounts in all tissues. These AD-MSC features seem to promote tissue repair while cell proliferation, angiogenesis, and anti-inflammatory processes are rapidly required in damaged sites [55]. In the management of mammary asymmetries by plastic and reconstructive surgeries, postoperative complications such as prolonged pain, hematomas, secondary cysts, infections, necrosis, capsular contracture, hypertrophic scars, and reintervention for prosthesis substitution were documented [56]. Major complications of microsurgical reconstruction of fingers such as lack of osseous integration of the implant, rare detachment of the prosthesis, or lack of acceptance by the patient were recorded as well [57]. Besides, in nasal reconstructive surgery, autologous bone grafts do not survive as well as cartilage grafts. Totality, satisfying esthetic results were achieved for both patient and surgeon in 79% of cases [58].

## Conclusions

Preconditioning diabetic AD-MSC with PBM in vitro significantly increased cell function compared with the control diabetic AD-MSC. PBM preconditioning of diabetic AD-MSC significantly increased healing in ischemic MRSA-infected delayed healing wound in rats with DM1 compared to the control, control AD-MSC, and AD-MSC plus PBM in vivo groups. The combined application of preconditioned diabetic AD-MSC with PBM in vitro plus PBM therapy in vivo demonstrated a significant superior effect compared to the other groups.

The cellular and molecular mechanisms of combined PBM and AD-MSC treatment on inflammation and proliferation steps of delayed wound healing of DM animals should be clarified by additional investigations. We also suggest further research with a combination of AD-MSC, PBM, and scaffolds or DS in diabetic subjects.

## Supplementary information

**Additional file 1.** Availability of Data and Materials

## Data Availability

Some more information about the results, as well as statistical analyses of in vitro test, tensiometrical, and wound closure rate, is provided in Additional file 1.

## Abbreviations

DM: Diabetes mellitus
DFU: Diabetic foot ulcer
MRSA: Methicillin-resistant *Staphylococcus aureus*
EPC: Endothelial progenitor cell
AD-MSCs: Adipose-derived mesenchymal stem cells
PBM: Photobiomodulation
HIF-1α: Hypoxia-inducible factor-1 alpha
DM2: Type two diabetes mellitus
MSC: Mesenchymal stem cell
DM1: Type 1 diabetic rats
PDT: Population doubling time
CFU: Colony-forming unit
IP: Intraperitoneal
STZ: Streptozotocin
CD: Cluster of differentiation markers
AO: Acridine orange
EB: Ethidium bromide
H&E: Hematoxylin and eosin
SD: Mean ± standard deviation
ANOVA: One-way analysis of variance
LSD: Least significant difference
AGEs: Advanced glycosylation end products
h: Human
HF: Hair follicle
ECM: Extracellular matrices
SVF: Stromal vascular fraction
PRP: Platelet-rich plasma
DS: Dermal substitute
HA: Hyaluronic acid
DRT: Dermal Regeneration Template
AD-SVFs: Adipose-derived stromal vascular fraction
CQAs: Critical quality attributes
DEG: Dermal epidermal graft
BM: Bone marrow

## References

[1] Atlas D (2015). International Diabetes Federation. IDF diabetes atlas.

[2] Saeedi P, Petersohn I, Salpea P, Malanda B, Karuranga S, Unwin N (2019). Global and regional diabetes prevalence estimates for 2019 and projections for 2030 and 2045: results from the International Diabetes Federation Diabetes Atlas, 9(th) edition. Diabetes Res Clin Pract.

[3] Cho N, Shaw J, Karuranga S, Huang Y, da Rocha FJ, Ohlrogge A (2018). IDF Diabetes Atlas: global estimates of diabetes prevalence for 2017 and projections for 2045. Diabetes Res Clin Pract.

[4] Alexiadou K, Doupis J (2012). Management of diabetic foot ulcers. Diabetes Ther.

[5] Dalla Paola L, Carone A, Ricci S, Russo A (2010). Advances in treatment of diabetic foot ulcers. Avances en Diabetología.

[6] Boulton AJ, Armstrong DG, Kirsner RS, Attinger CE, Lavery LA, Lipsky BA (2018). Diagnosis and management of diabetic foot complications.

[7] Eleftheriadou I, Tentolouris N, Argiana V, Jude E, Boulton AJ (2010). Methicillin-resistant *Staphylococcus aureus* in diabetic foot infections. Drugs..

[8] Lipsky BA (2016). Diabetic foot infections: current treatment and delaying the ‘post-antibiotic era’. Diabetes Metab Res Rev.

[9] Baltzis D, Eleftheriadou I, Veves A (2014). Pathogenesis and treatment of impaired wound healing in diabetes mellitus: new insights. Adv Ther.

[10] Clayton W, Elasy TA (2009). A review of the pathophysiology, classification, and treatment of foot ulcers in diabetic patients. Clin Diab.

[11] Davis FM, Kimball A, Boniakowski A, Gallagher K (2018). Dysfunctional wound healing in diabetic foot ulcers: new crossroads. Curr Diab Rep.

[12] Catrina SB, Zheng X (2016). Disturbed hypoxic responses as a pathogenic mechanism of diabetic foot ulcers. Diabetes Metab Res Rev.

[13] Jeffcoate WJ, Vileikyte L, Boyko EJ, Armstrong DG, Boulton AJM (2018). Current challenges and opportunities in the prevention and management of diabetic foot ulcers. Diabetes Care.

[14] Gadelkarim M, Abushouk AI, Ghanem E, Hamaad AM, Saad AM, Abdel-Daim MM (2018). Adipose-derived stem cells: effectiveness and advances in delivery in diabetic wound healing. Biomed Pharmacother.

[15] Sumi M, Sata M, Toya N, Yanaga K, Ohki T, Nagai R (2007). Transplantation of adipose stromal cells, but not mature adipocytes, augments ischemia-induced angiogenesis. Life Sci.

[16] Burdon TJ, Paul A, Noiseux N, Prakash S, Shum-Tim D. Bone marrow stem cell derived paracrine factors for regenerative medicine: current perspectives and therapeutic potential. Bone Marrow Res. 2011;2011:207326. 10.1155/2011/207326. 10.1155/2011/207326 PMC3195349 22046556

[17] Cerqueira MT, Pirraco RP, Marques AP (2016). Stem cells in skin wound healing: are we there yet?. Adv Wound Care.

[18] van de Vyver M (2017). Intrinsic mesenchymal stem cell dysfunction in diabetes mellitus: implications for autologous cell therapy. Stem Cells Dev.

[19] Shin L, Peterson DA (2012). Impaired therapeutic capacity of autologous stem cells in a model of type 2 diabetes. Stem Cells Transl Med.

[20] Hamblin MR (2017). Mechanisms and applications of the anti-inflammatory effects of photobiomodulation. AIMS Biophys.

[21] Amini A, Pouriran R, Abdollahifar M-A, Abbaszadeh HA, Ghoreishi SK, Chien S (2018). Stereological and molecular studies on the combined effects of photobiomodulation and human bone marrow mesenchymal stem cell conditioned medium on wound healing in diabetic rats. J Photochem Photobiol B Biol.

[22] Cury V, Moretti AIS, Assis L, Bossini P, de Souza CJ, Neto CB (2013). Low level laser therapy increases angiogenesis in a model of ischemic skin flap in rats mediated by VEGF, HIF-1α and MMP-2. J Photochem Photobiol B Biol.

[23] Moradi A, Zare F, Mostafavinia A, Safaju S, Shahbazi A, Habibi M (2020). Photobiomodulation plus adipose-derived stem cells improve healing of ischemic infected wounds in type 2 diabetic rats. Sci Rep.

[24] AlGhamdi KM, Kumar A, Moussa NA (2012). Low-level laser therapy: a useful technique for enhancing the proliferation of various cultured cells. Lasers Med Sci.

[25] Liu Y, Zhang H (2016). Low-level laser irradiation precondition for cardiac regenerative therapy. Photomed Laser Surg.

[26] Ebrahimpour-Malekshah R, Amini A, Zare F, Mostafavinia A, Davoody S, Deravi N, Rahmanian M, Hashemi SM, Habibi M, Ghoreishi SK, Chien S, Shafikhani S, Ahmadi H, Bayat S, Bayat M. Combined therapy of photobiomodulation and adipose-derived stem cells synergistically improve healing in an ischemic, infected and delayed healing wound model in rats with type 1 diabetes mellitus. BMJ Open Diabetes Res Care. 2020;8(1):e001033. 10.1136/bmjdrc-2019-001033. PMID: 32098898; PMCID: PMC7206914. 10.1136/bmjdrc-2019-001033 PMC7206914 32098898

[27] Liao X, Li SH, Xie GH, Xie S, Xiao LL, Song JX (2018). Preconditioning with low-level laser irradiation enhances the therapeutic potential of human adipose-derived stem cells in a mouse model of photoaged skin. Photochem Photobiol.

[28] Muhammad G, Xu J, Bulte JWM, Jablonska A, Walczak P, Janowski M (2017). Transplanted adipose-derived stem cells can be short-lived yet accelerate healing of acid-burn skin wounds: a multimodal imaging study. Sci Rep.

[29] Zare F, Moradi A, Fallahnezhad S, Ghoreishi SK, Amini A, Chien S (2019). Photobiomodulation with 630 plus 810nm wavelengths induce more in vitro cell viability of human adipose stem cells than human bone marrow-derived stem cells. J Photochem Photobiol B.

[30] Dyson M, Young SR, Hart J, Lynch JA, Lang S (1992). Comparison of the effects of moist and dry conditions on the process of angiogenesis during dermal repair. J Invest Dermatol.

[31] Young SR, Dyson M (1990). Effect of therapeutic ultrasound on the healing of full-thickness excised skin lesions. Ultrasonics..

[32] Cañedo-Dorantes L, Cañedo-Ayala M. Skin Acute Wound Healing: A Comprehensive Review. Int J Inflam. 2019;2019:3706315. 10.1155/2019/3706315. PMID: 31275545; PMCID: PMC6582859. 10.1155/2019/3706315 PMC6582859 31275545

[33] Chouhan D, Dey N, Bhardwaj N, Mandal BB. Emerging and innovative approaches for wound healing and skin regeneration: current status and advances. Biomaterials. 2019;216:119267. 10.1016/j.biomaterials.2019.119267. 10.1016/j.biomaterials.2019.119267 31247480

[34] Dehkordi AN, Babaheydari FM, Chehelgerdi M, Dehkordi SR (2019). Skin tissue engineering: wound healing based on stem-cell-based therapeutic strategies. Stem Cell Res Ther.

[35] Shingyochi Y, Orbay H, Mizuno H (2015). Adipose-derived stem cells for wound repair and regeneration. Expert Opin Biol Ther.

[36] Chen X, Yan L, Guo Z, Chen Z, Chen Y, Li M (2016). Adipose-derived mesenchymal stem cells promote the survival of fat grafts via crosstalk between the Nrf2 and TLR4 pathways. Cell Death Dis.

[37] Lopes L, Setia O, Aurshina A, Liu S, Hu H, Isaji T (2018). Stem cell therapy for diabetic foot ulcers: a review of preclinical and clinical research. Stem Cell Res Ther.

[38] Gong JH, Dong JY, Xie T, Lu SL (2017). The influence of AGEs environment on proliferation, apoptosis, homeostasis, and endothelial cell differentiation of human adipose stem cells. Int J Low Extrem Wounds.

[39] Abdi J, Rashedi I, Keating A (2018). Concise review: TLR pathway-miRNA interplay in mesenchymal stromal cells: regulatory roles and therapeutic directions. Stem Cells.

[40] Pugin J (2012). How tissue injury alarms the immune system and causes a systemic inflammatory response syndrome. Ann Intensive Care.

[41] Zampell JC, Yan A, Avraham T, Andrade V, Malliaris S, Aschen S (2011). Temporal and spatial patterns of endogenous danger signal expression after wound healing and in response to lymphedema. Am J Phys Cell Phys.

[42] Basith S, Manavalan B, Yoo TH, Kim SG, Choi S (2012). Roles of toll-like receptors in cancer: a double-edged sword for defense and offense. Arch Pharm Res.

[43] Julier Z, Park AJ, Briquez PS, Martino MM (2017). Promoting tissue regeneration by modulating the immune system. Acta Biomater.

[44] Lin W, Xu L, Zwingenberger S, Gibon E, Goodman SB, Li G (2017). Mesenchymal stem cells homing to improve bone healing. J Orthop Transl.

[45] Wu Y, Chen L, Scott PG, Tredget EE. Mesenchymal stem cells enhance wound healing through differentiation and angiogenesis. Stem Cells. 2007;25(10):2648–59. 10.1634/stemcells.2007-0226. Epub 2007 Jul 5. PMID: 17615264. 10.1634/stemcells.2007-0226 17615264

[46] Li P, Guo X (2018). A review: therapeutic potential of adipose-derived stem cells in cutaneous wound healing and regeneration. Stem Cell Res Ther.

[47] Kushibiki T, Hirasawa T, Okawa S, Ishihara M. Low reactive level laser therapy for mesenchymal stromal cells therapies. Stem Cells Int. 2018;9(1):302. 10.1186/s13287-018-1044-5. 10.1155/2015/974864 PMC4529981 26273309

[48] Houschyar KS, Borrelli MR, Tapking C, Popp D, Puladi B, Ooms M (2020). Molecular mechanisms of hair growth and regeneration: current understanding and novel paradigms. Dermatology (Basel, Switzerland).

[49] Ansell DM, Kloepper JE, Thomason HA, Paus R, Hardman MJ (2011). Exploring the “hair growth-wound healing connection”: anagen phase promotes wound re-epithelialization. J Invest Dermatol.

[50] Mosca RC, Ong AA, Albasha O, Bass K, Arany P (2019). Photobiomodulation therapy for wound care: a potent, noninvasive, photoceutical approach. Adv Skin Wound Care.

[51] Coalson E, Bishop E, Liu W, Feng Y, Spezia M, Liu B (2019). Stem cell therapy for chronic skin wounds in the era of personalized medicine: from bench to bedside. Genes Dis.

[52] Tamama K, Kerpedjieva SS (2012). Acceleration of wound healing by multiple growth factors and cytokines secreted from multipotential stromal cells/mesenchymal stem cells. Adv Wound Care.

[53] Sen CK. Human wounds and its burden: an updated compendium of estimates. Adv Wound Care (New Rochelle). 2019;8(2):39–48. 10.1089/wound.2019.0946. 10.1089/wound.2019.0946 PMC6389759 30809421

[54] Rezai Rad M, Bohloli M, Akhavan Rahnama M, Anbarlou A, Nazeman P, Khojasteh A. Impact of tissue harvesting sites on the cellular behaviors of adipose-derived stem cells: implication for bone tissue engineering. Stem Cells Int. 2017;2017:2156478. 10.1155/2017/2156478. Epub 2017 Dec 14. PMID: 29387089; PMCID: PMC5745705. 10.1155/2017/2156478 PMC5745705 29387089

[55] Mazini L, Rochette L, Amine M, Malka G. Regenerative capacity of Adipose Derived Stem Cells (ADSCs), comparison with Mesenchymal Stem Cells (MSCs). Int J Mol Sci. 2019 May 22;20(10):2523. 10.3390/ijms20102523. PMID: 31121953; PMCID: PMC6566837. 10.3390/ijms20102523 PMC6566837 31121953

[56] Araco A, Gravante G, Araco F, Gentile P, Castri F, Delogu D (2006). Breast asymmetries: a brief review and our experience. Aesthet Plast Surg.

[57] Cervelli V, Bottini DJ, Arpino A, Grimaldi M, Rogliani M, Gentile P. Bone-anchored implant in cosmetic finger reconstruction. Ann Chir Plast Esthet. Elsevier; 2008;53(4):365–7. 10.1016/j.anplas.2007.06.010. 10.1016/j.anplas.2007.06.010 18031918

[58] Bottini D, Gentile P, Donfrancesco A, Fiumara L, Cervelli V (2008). Augmentation rhinoplasty with autologous grafts. Aesthet Plast Surg.

