# Supplementary material for: Transplantation of photobiomodulation-preconditioned diabetic stem cells accelerates ischemic wound healing in diabetic rats
Source: Stem Cell Res Ther. 2020 Nov 25;11:494. doi: 10.1186/s13287-020-01967-2 (PMC7688005; doi:10.1186/s13287-020-01967-2)
Supplement: Supplementary file 1 — Additional file 1. Availability of Data and Materials [file 13287_2020_1967_MOESM1_ESM.docx]

| Figure 7↓.  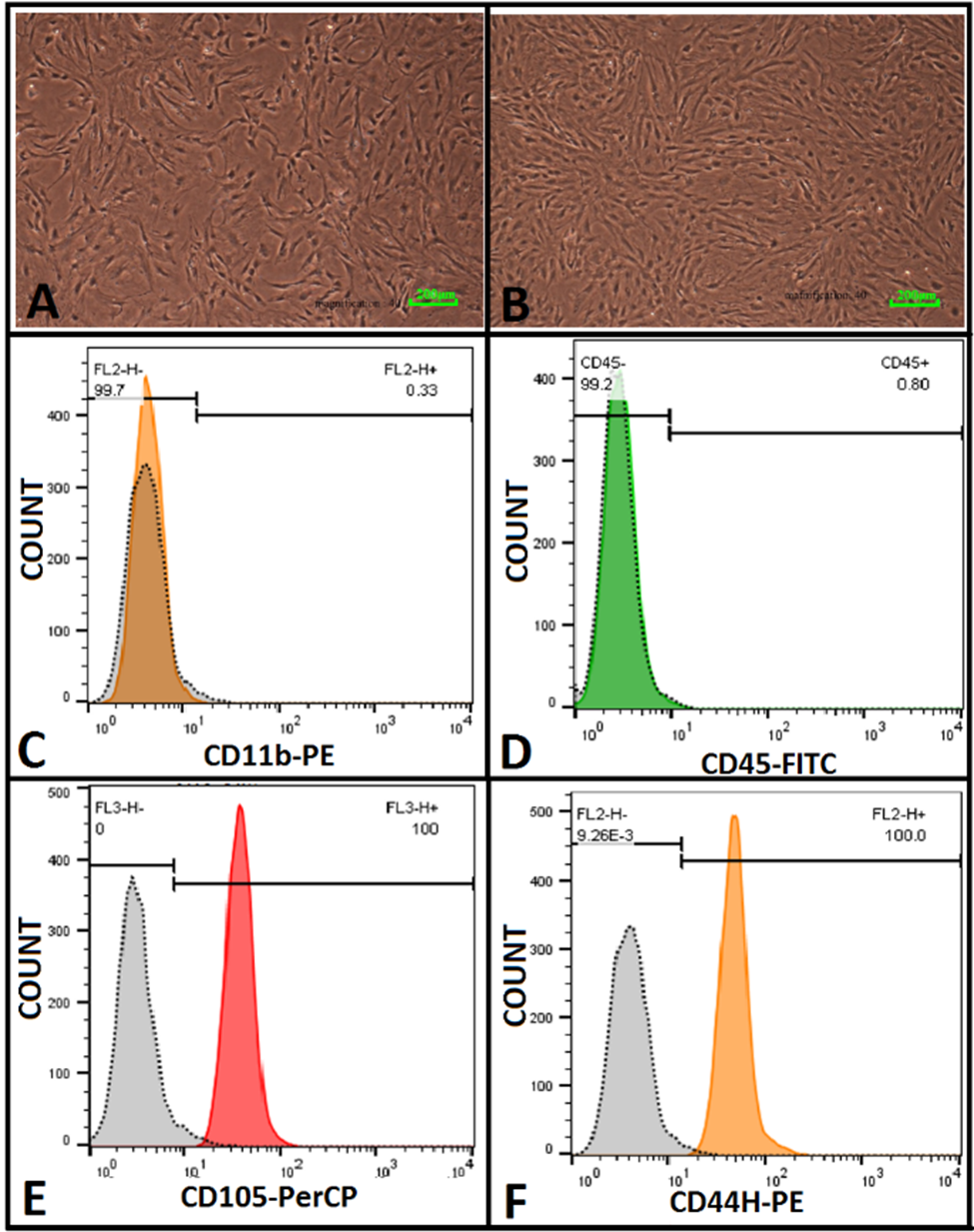  **Figure 7. Passage-4 adipose-derived mesenchymal Stem Cells (AD-MSC) of the control diabetic ADS (panel A) and experimental diabetic AD-MSC (panel B) and immunophenotypes of the AD-MSC are shown in panels C (CD11b), D (CD45), E (CD105), and F (CD44H).**  Table 2. Comparison of body weights and blood sugar values (mean ±SD) of the studied groups in the first and second phases of the experiment.   \| **GROUPS** \| **CONTROL** \| **CONTROL**  **AD-MSC** \| **AD-MSC +PBM**  **IN VIVO** \| **AD-MSC +PBM**  **IN VITRO** \| **AD-MSC +PBM**  **IN VITRO+IN VIVO** \| \| --- \| --- \| --- \| --- \| --- \| --- \| \| **PHASES 1, 2↓** \| \| **FACTORS** \| \| **1. Initial blood glucose (mg/dl)** \| 452.5±73.82 \| – \| – \| – \| – \| \| **Final blood glucose (mg/dl)** \| 457.75±74.07 \| \| **1. Initial body weight (g)** \| 264.9±22.49 \| – \| – \| – \| – \| \| **Final body weight (g)** \| 230.6±16.63** \| \| **2. Initial blood glucose (mg/dl)** \| 479.6±54 \| 345.2±82.41 \| 393.42±101.08 \| 479.6±54.01 \| 425.16±27.3 \| \| **2. Final blood glucose (mg/dl)** \| 565.8±60.69*** \| 408.2±82.26*** \| 355.57±89.05 \| 453±135.42 \| 445±24.60** \| \| **2. Initial body weight (g)** \| 294.4±36.44 \| 300.8±13.14 \| 303.28±9.34 \| 303.6±19.034 \| 295.5±19.29 \| \| **2. Final body weight (g)** \| 232±32.43*** \| 267±24.41* \| 294±20.51 \| 268.6±19.165*** \| 277.17±18.90*** \|   *p<0.05; **p<0.01; ***p<0.001 ;PBM: Photobiomodulation; AD-MSC: adipose-derived stem cell.  Figure 8↓.  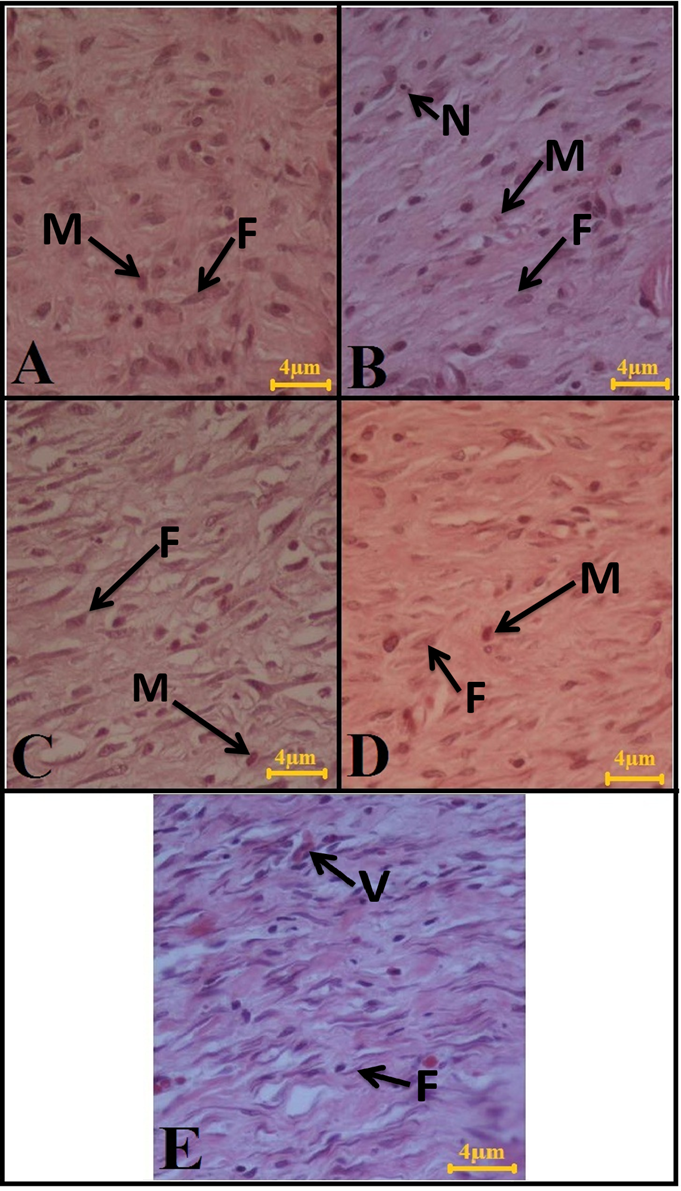  **Figure 8. Wound tissue stained with Hematoxylin and eosin method for demonstrating neutrophils, macrophages, fibroblasts, and blood vessels of the studied groups (A, control group; B, control- adipose-derived stem cell (ADS) ; C, ADS+ photobiomodulation(PBM) in vivo; D, ADS+PBM- in vitro; E, ADS+PBM –invitro+ in vivo.** F: Fibroblast; M: Macrophage; N: Neutrophil; V: Blood vessel.  **Figure 9↓.**  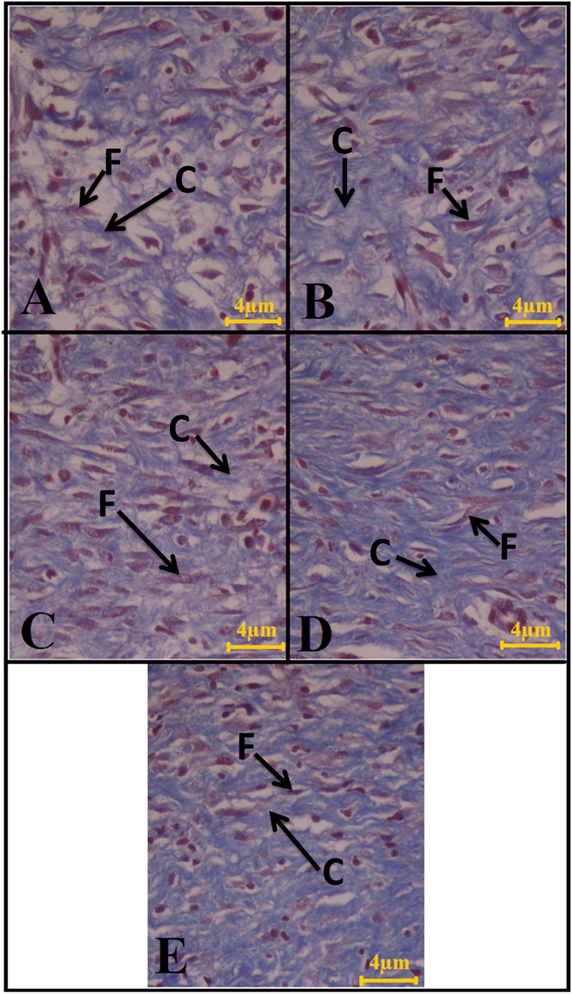  **Figure 9. Wound tissue stained with Mallory's trichrome staining method for demonstrating collagen fibers of the studied groups (A, control group; B, control- adipose-derived stem cell (ADS) ; C, ADS+ photobiomodulation(PBM) in vivo; D, ADS+PBM- in vitro; E, ADS+PBM –invitro+ in vivo. Histological photos of the wound area as seen by Mallory's trichrome staining in the five studied groups on day 16 were shown in below figure.** Collagen fibers are visualized with the blue color.  **In vitro analysis, MTT test**  **Case Summaries** | | | | |
| --- | --- | --- | --- | --- | --- | --- | --- | --- | --- | --- | --- | --- | --- | --- | --- | --- | --- | --- | --- | --- | --- | --- | --- | --- | --- | --- | --- | --- | --- | --- | --- | --- | --- | --- | --- | --- | --- | --- | --- | --- | --- | --- | --- | --- | --- | --- | --- | --- | --- | --- | --- | --- |
| OD | | | | |
| Dose | Groups | Mean | Std. Deviation | Std. Error of Mean |
| 1.2 JOULE | HEALTHY CONTROL | 1.3567 | .04041 | .02333 |
|  | DIABETIC CONTROL | .9833 | .01528 | .00882 |
|  | DIABETIC EXPRIMENTAL | 1.9833 | .02082 | .01202 |
| 2.4 JOULE | HEALTHY CONTROL | 1.1633 | .05132 | .02963 |
|  | DIABETIC CONTROL | .9467 | .05132 | .02963 |
|  | DIABETIC EXPRIMENTAL | 1.7233 | .06807 | .03930 |

**Dose = 1.2 JOULE**

| **ANOVA^a^** | | | | | |
| --- | --- | --- | --- | --- | --- |
| OD | | | | | |
|  | Sum of Squares | df | Mean Square | F | Sig. |
| Between Groups | 1.532 | 2 | .766 | 999.188 | .000 |
| Within Groups | .005 | 6 | .001 |  |  |
| Total | 1.537 | 8 |  |  |  |
| a. Dose = 1.2 JOULE | | | | | |

**Post Hoc Tests**

| (I) Groups | (J) Groups | Mean Difference (I-J) | Std. Error | Sig. |
| --- | --- | --- | --- | --- |
|  |  |  |  |  |
| HEALTHY CONTROL | DIABETIC CONTROL | .37333^*^ | .02261 | .000 |
|  | DIABETIC EXPRIMENTAL | -.62667^*^ | .02261 | .000 |
| DIABETIC CONTROL | HEALTHY CONTROL | -.37333^*^ | .02261 | .000 |
|  | DIABETIC EXPRIMENTAL | -1.00000^*^ | .02261 | .000 |
| DIABETIC EXPRIMENTAL | HEALTHY CONTROL | .62667^*^ | .02261 | .000 |
|  | DIABETIC CONTROL | 1.00000^*^ | .02261 | .000 |

**Dose = 2.4 JOULE**

| **ANOVA^a^** | | | | | |
| --- | --- | --- | --- | --- | --- |
| OD | | | | | |
|  | Sum of Squares | df | Mean Square | F | Sig. |
| Between Groups | .964 | 2 | .482 | 146.024 | .000 |
| Within Groups | .020 | 6 | .003 |  |  |
| Total | .984 | 8 |  |  |  |
| a. Dose = 2.4 JOULE | | | | | |

**Post Hoc Tests**

| (I) Groups | (J) Groups | Mean Difference (I-J) | Std. Error | Sig. |
| --- | --- | --- | --- | --- |
|  |  |  |  |  |
| HEALTHY CONTROL | DIABETIC CONTROL | .21667^*^ | .04690 | .004 |
|  | DIABETIC EXPRIMENTAL | -.56000^*^ | .04690 | .000 |
| DIABETIC CONTROL | HEALTHY CONTROL | -.21667^*^ | .04690 | .004 |
|  | DIABETIC EXPRIMENTAL | -.77667^*^ | .04690 | .000 |
| DIABETIC EXPRIMENTAL | HEALTHY CONTROL | .56000^*^ | .04690 | .000 |
|  | DIABETIC CONTROL | .77667^*^ | .04690 | .000 |

| **Case Summaries** | | | | |
| --- | --- | --- | --- | --- |
| PDT | | | | |
| Dose | Groups | Mean | Std. Deviation | Std. Error of Mean |
| 1.2 JOULE | HEALTHY CONTROL | 43.6167 | 4.10822 | 2.37188 |
|  | DIABETIC CONTROL | 67.7900 | 2.50944 | 1.44883 |
|  | DIABETIC EXPRIMENTAL | 46.1867 | 2.74404 | 1.58427 |
| 2.4 JOULE | HEALTHY CONTROL | 41.2000 | .98549 | .56898 |
|  | DIABETIC CONTROL | 68.4667 | 2.07849 | 1.20002 |
|  | DIABETIC EXPRIMENTAL | 44.0833 | 2.13664 | 1.23359 |

**Dose = 1.2 JOULE**

| **ANOVA^a^** | | | | | |
| --- | --- | --- | --- | --- | --- |
| PD | | | | | |
|  | Sum of Squares | df | Mean Square | F | Sig. |
| Between Groups | 1057.659 | 2 | 528.829 | 51.670 | .000 |
| Within Groups | 61.409 | 6 | 10.235 |  |  |
| Total | 1119.068 | 8 |  |  |  |
| a. Dose = 1.2 JOULE | | | | | |

**Post Hoc Tests**

| (I) Groups | (J) Groups | Mean Difference (I-J) | Std. Error | Sig. |
| --- | --- | --- | --- | --- |
|  |  |  |  |  |
| HEALTHY CONTROL | DIABETIC CONTROL | -24.17333^*^ | 2.61213 | .000 |
|  | DIABETIC EXPRIMENTAL | -2.57000 | 2.61213 | .363 |
| DIABETIC CONTROL | HEALTHY CONTROL | 24.17333^*^ | 2.61213 | .000 |
|  | DIABETIC EXPRIMENTAL | 21.60333^*^ | 2.61213 | .000 |
| DIABETIC EXPRIMENTAL | HEALTHY CONTROL | 2.57000 | 2.61213 | .363 |
|  | DIABETIC CONTROL | -21.60333^*^ | 2.61213 | .000 |

**Dose = 2.4 JOULE**

| **ANOVA^a^** | | | | | |
| --- | --- | --- | --- | --- | --- |
| PD | | | | | |
|  | Sum of Squares | df | Mean Square | F | Sig. |
| Between Groups | 1346.332 | 2 | 673.166 | 204.889 | .000 |
| Within Groups | 19.713 | 6 | 3.286 |  |  |
| Total | 1366.045 | 8 |  |  |  |
| a. Dose = 2.4 JOULE | | | | | |

**Post Hoc Tests**

| (I) Groups | (J) Groups | Mean Difference (I-J) | Std. Error | Sig. |
| --- | --- | --- | --- | --- |
|  |  |  |  |  |
| HEALTHY CONTROL | DIABETIC CONTROL | -27.26667^*^ | 1.47998 | .000 |
|  | DIABETIC EXPRIMENTAL | -2.88333 | 1.47998 | .099 |
| DIABETIC CONTROL | HEALTHY CONTROL | 27.26667^*^ | 1.47998 | .000 |
|  | DIABETIC EXPRIMENTAL | 24.38333^*^ | 1.47998 | .000 |
| DIABETIC EXPRIMENTAL | HEALTHY CONTROL | 2.88333 | 1.47998 | .099 |
|  | DIABETIC CONTROL | -24.38333^*^ | 1.47998 | .000 |

| **Case Summaries** | | | | |
| --- | --- | --- | --- | --- |
| APOPTOSIS | | | | |
| Dose | Groups | Mean | Std. Deviation | Std. Error of Mean |
| 1.2 JOULE | HEALTHY CONTROL | 3.1700 | .22338 | .12897 |
|  | DIABETIC CONTROL | 6.6033 | .72390 | .41794 |
|  | DIABETIC EXPRIMENTAL | 2.9767 | .82597 | .47688 |
| 2.4 JOULE | HEALTHY CONTROL | 1.9400 | .68790 | .39716 |
|  | DIABETIC CONTROL | 3.8967 | .38423 | .22184 |
|  | DIABETIC EXPRIMENTAL | 2.0300 | .45255 | .32000 |

**Dose = 1.2 JOULE**

| **ANOVA^a^** | | | | | |
| --- | --- | --- | --- | --- | --- |
| APOPTOSIS | | | | | |
|  | Sum of Squares | df | Mean Square | F | Sig. |
| Between Groups | 24.978 | 2 | 12.489 | 29.826 | .001 |
| Within Groups | 2.512 | 6 | .419 |  |  |
| Total | 27.490 | 8 |  |  |  |
| a. Dose = 1.2 JOULE | | | | | |

**Post Hoc Tests**

| (I) Groups | (J) Groups | Mean Difference (I-J) | Std. Error | Sig. |
| --- | --- | --- | --- | --- |
|  |  |  |  |  |
| HEALTHY CONTROL | DIABETIC CONTROL | -3.43333^*^ | .52834 | .001 |
|  | DIABETIC EXPRIMENTAL | .19333 | .52834 | .727 |
| DIABETIC CONTROL | HEALTHY CONTROL | 3.43333^*^ | .52834 | .001 |
|  | DIABETIC EXPRIMENTAL | 3.62667^*^ | .52834 | .000 |
| DIABETIC EXPRIMENTAL | HEALTHY CONTROL | -.19333 | .52834 | .727 |
|  | DIABETIC CONTROL | -3.62667^*^ | .52834 | .000 |

**Dose = 2.4 JOULE**

| **ANOVA^a^** | | | | | |
| --- | --- | --- | --- | --- | --- |
| APOPTOSIS | | | | | |
|  | Sum of Squares | df | Mean Square | F | Sig. |
| Between Groups | 6.927 | 2 | 3.463 | 11.971 | .012 |
| Within Groups | 1.446 | 5 | .289 |  |  |
| Total | 8.373 | 7 |  |  |  |
| a. Dose = 2.4 JOULE | | | | | |

**Post Hoc Tests**

| (I) Groups | (J) Groups | Mean Difference (I-J) | Std. Error | Sig. |
| --- | --- | --- | --- | --- |
|  |  |  |  |  |
| HEALTHY CONTROL | DIABETIC CONTROL | -1.95667^*^ | .43916 | .007 |
|  | DIABETIC EXPRIMENTAL | -.09000 | .49100 | .862 |
| DIABETIC CONTROL | HEALTHY CONTROL | 1.95667^*^ | .43916 | .007 |
|  | DIABETIC EXPRIMENTAL | 1.86667^*^ | .49100 | .013 |
| DIABETIC EXPRIMENTAL | HEALTHY CONTROL | .09000 | .49100 | .862 |
|  | DIABETIC CONTROL | -1.86667^*^ | .49100 | .013 |

| **Tensiometrical Analysis**  **Case Summaries** | | | | | |
| --- | --- | --- | --- | --- | --- |
| Group | | Force | Stress | Energy | Bending |
| CONTROL | Mean | 1.1400 | .1140 | 1.8880 | 1.1180 |
|  | Std. Deviation | .13077 | .01308 | .31011 | .13293 |
|  | Std. Error of Mean | .05848 | .00585 | .13869 | .05945 |
| CONTROL(ADS) | Mean | 3.0386 | .3039 | 7.2128 | 6.6403 |
|  | Std. Deviation | .27631 | .02763 | 1.38120 | .60838 |
|  | Std. Error of Mean | .12357 | .01236 | .61769 | .27207 |
| PBM+ADS(Invivo) | Mean | 3.1600 | .3160 | 13.9600 | 6.1800 |
|  | Std. Deviation | .51769 | .05177 | 1.42232 | .63797 |
|  | Std. Error of Mean | .23152 | .02315 | .63608 | .28531 |
| PBM+ADS(Invitro) | Mean | 5.5754 | .5575 | 16.4434 | 9.0196 |
|  | Std. Deviation | .46859 | .04686 | 1.79725 | 1.16510 |
|  | Std. Error of Mean | .20956 | .02096 | .80375 | .52105 |
| PBM+ADS(Invivo&Invitro) | Mean | 8.1167 | .8117 | 38.8167 | 12.8517 |
|  | Std. Deviation | .84951 | .08495 | 5.24840 | 1.14892 |
|  | Std. Error of Mean | .34681 | .03468 | 2.14265 | .46904 |
| Total | Mean | 4.3565 | .4357 | 16.5547 | 7.3807 |
|  | Std. Deviation | 2.57500 | .25750 | 13.69082 | 4.07080 |
|  | Std. Error of Mean | .50500 | .05050 | 2.68499 | .79835 |

| **ANOVA** | | | | | | |
| --- | --- | --- | --- | --- | --- | --- |
|  | | Sum of Squares | df | Mean Square | F | Sig. |
| Force | Between Groups | 159.833 | 4 | 39.958 | 141.447 | .000 |
|  | Within Groups | 5.932 | 21 | .282 |  |  |
|  | Total | 165.766 | 25 |  |  |  |
| Stress | Between Groups | 1.598 | 4 | .400 | 141.447 | .000 |
|  | Within Groups | .059 | 21 | .003 |  |  |
|  | Total | 1.658 | 25 |  |  |  |
| Energy | Between Groups | 4519.211 | 4 | 1129.803 | 142.279 | .000 |
|  | Within Groups | 166.756 | 21 | 7.941 |  |  |
|  | Total | 4685.967 | 25 |  |  |  |
| Bending | Between Groups | 399.076 | 4 | 99.769 | 137.756 | .000 |
|  | Within Groups | 15.209 | 21 | .724 |  |  |
|  | Total | 414.285 | 25 |  |  |  |

**Post Hoc Tests**

| Dependent Variable | (I) Group | (J) Group | Sig. |
| --- | --- | --- | --- |
|  |  |  |  |
| Force | CONTROL | CONTROL(ADS) | .000 |
|  |  | PBM+ADS(Invivo) | .000 |
|  |  | PBM+ADS(Invitro) | .000 |
|  |  | PBM+ADS(Invivo&Invitro) | .000 |
|  | CONTROL(ADS) | CONTROL | .000 |
|  |  | PBM+ADS(Invivo) | .722 |
|  |  | PBM+ADS(Invitro) | .000 |
|  |  | PBM+ADS(Invivo&Invitro) | .000 |
|  | PBM+ADS(Invivo) | CONTROL | .000 |
|  |  | CONTROL(ADS) | .722 |
|  |  | PBM+ADS(Invitro) | .000 |
|  |  | PBM+ADS(Invivo&Invitro) | .000 |
|  | PBM+ADS(Invitro) | CONTROL | .000 |
|  |  | CONTROL(ADS) | .000 |
|  |  | PBM+ADS(Invivo) | .000 |
|  |  | PBM+ADS(Invivo&Invitro) | .000 |
|  | PBM+ADS(Invivo&Invitro) | CONTROL | .000 |
|  |  | CONTROL(ADS) | .000 |
|  |  | PBM+ADS(Invivo) | .000 |
|  |  | PBM+ADS(Invitro) | .000 |
| Stress | CONTROL | CONTROL(ADS) | .000 |
|  |  | PBM+ADS(Invivo) | .000 |
|  |  | PBM+ADS(Invitro) | .000 |
|  |  | PBM+ADS(Invivo&Invitro) | .000 |
|  | CONTROL(ADS) | CONTROL | .000 |
|  |  | PBM+ADS(Invivo) | .722 |
|  |  | PBM+ADS(Invitro) | .000 |
|  |  | PBM+ADS(Invivo&Invitro) | .000 |
|  | PBM+ADS(Invivo) | CONTROL | .000 |
|  |  | CONTROL(ADS) | .722 |
|  |  | PBM+ADS(Invitro) | .000 |
|  |  | PBM+ADS(Invivo&Invitro) | .000 |
|  | PBM+ADS(Invitro) | CONTROL | .000 |
|  |  | CONTROL(ADS) | .000 |
|  |  | PBM+ADS(Invivo) | .000 |
|  |  | PBM+ADS(Invivo&Invitro) | .000 |
|  | PBM+ADS(Invivo&Invitro) | CONTROL | .000 |
|  |  | CONTROL(ADS) | .000 |
|  |  | PBM+ADS(Invivo) | .000 |
|  |  | PBM+ADS(Invitro) | .000 |
| Energy | CONTROL | CONTROL(ADS) | .007 |
|  |  | PBM+ADS(Invivo) | .000 |
|  |  | PBM+ADS(Invitro) | .000 |
|  |  | PBM+ADS(Invivo&Invitro) | .000 |
|  | CONTROL(ADS) | CONTROL | .007 |
|  |  | PBM+ADS(Invivo) | .001 |
|  |  | PBM+ADS(Invitro) | .000 |
|  |  | PBM+ADS(Invivo&Invitro) | .000 |
|  | PBM+ADS(Invivo) | CONTROL | .000 |
|  |  | CONTROL(ADS) | .001 |
|  |  | PBM+ADS(Invitro) | .178 |
|  |  | PBM+ADS(Invivo&Invitro) | .000 |
|  | PBM+ADS(Invitro) | CONTROL | .000 |
|  |  | CONTROL(ADS) | .000 |
|  |  | PBM+ADS(Invivo) | .178 |
|  |  | PBM+ADS(Invivo&Invitro) | .000 |
|  | PBM+ADS(Invivo&Invitro) | CONTROL | .000 |
|  |  | CONTROL(ADS) | .000 |
|  |  | PBM+ADS(Invivo) | .000 |
|  |  | PBM+ADS(Invitro) | .000 |
| Bending | CONTROL | CONTROL(ADS) | .000 |
|  |  | PBM+ADS(Invivo) | .000 |
|  |  | PBM+ADS(Invitro) | .000 |
|  |  | PBM+ADS(Invivo&Invitro) | .000 |
|  | CONTROL(ADS) | CONTROL | .000 |
|  |  | PBM+ADS(Invivo) | .402 |
|  |  | PBM+ADS(Invitro) | .000 |
|  |  | PBM+ADS(Invivo&Invitro) | .000 |
|  | PBM+ADS(Invivo) | CONTROL | .000 |
|  |  | CONTROL(ADS) | .402 |
|  |  | PBM+ADS(Invitro) | .000 |
|  |  | PBM+ADS(Invivo&Invitro) | .000 |
|  | PBM+ADS(Invitro) | CONTROL | .000 |
|  |  | CONTROL(ADS) | .000 |
|  |  | PBM+ADS(Invivo) | .000 |
|  |  | PBM+ADS(Invivo&Invitro) | .000 |
|  | PBM+ADS(Invivo&Invitro) | CONTROL | .000 |
|  |  | CONTROL(ADS) | .000 |
|  |  | PBM+ADS(Invivo) | .000 |
|  |  | PBM+ADS(Invitro) | .000 |

| **Wound closure rate**  **Case Summaries** | | | | | |
| --- | --- | --- | --- | --- | --- |
| Group | | ULCER.RATE.4 | ULCER.RATE.8 | ULCER.RATE.12 | ULCER.RATE.16 |
| CONTROL | Mean | 1.9444 | 40.8631 | 76.4286 | 92.9122 |
|  | Std. Deviation | .51660 | 2.20299 | 3.93774 | 1.59362 |
|  | Std. Error of Mean | .21090 | .89937 | 1.96887 | .79681 |
| CONTROL(ADS) | Mean | 1.5242 | 50.0169 | 82.2232 | 95.5929 |
|  | Std. Deviation | .28725 | 4.32854 | 5.60125 | 2.71035 |
|  | Std. Error of Mean | .10857 | 1.63603 | 2.11707 | 1.21210 |
| PBM+ADS(Invivo) | Mean | 1.0235 | 54.8941 | 88.2471 | 96.9882 |
|  | Std. Deviation | .17054 | 2.36994 | 1.63409 | 3.67715 |
|  | Std. Error of Mean | .07627 | 1.05987 | .73079 | 1.64447 |
| PBM+ADS(Invitro) | Mean | 1.4941 | 54.1882 | 86.9529 | 97.3435 |
|  | Std. Deviation | .23749 | 2.31155 | 2.19917 | 3.49321 |
|  | Std. Error of Mean | .10621 | 1.03376 | .98350 | 1.56221 |
| PBM+ADS(Invivo&Invitro) | Mean | 1.4133 | 60.9162 | 88.8548 | 99.3402 |
|  | Std. Deviation | .28833 | 1.76978 | 1.09693 | 1.02231 |
|  | Std. Error of Mean | .11771 | .72251 | .44782 | .41736 |
| Total | Mean | 1.4967 | 51.9381 | 84.8298 | 96.6925 |
|  | Std. Deviation | .42073 | 7.34506 | 5.47307 | 3.24647 |
|  | Std. Error of Mean | .07813 | 1.36394 | 1.05329 | .64929 |

| **ANOVA** | | | | | | |
| --- | --- | --- | --- | --- | --- | --- |
|  | | Sum of Squares | df | Mean Square | F | Sig. |
| ULCER.RATE.4 | Between Groups | 2.369 | 4 | .592 | 5.495 | .003 |
|  | Within Groups | 2.587 | 24 | .108 |  |  |
|  | Total | 4.956 | 28 |  |  |  |
| ULCER.RATE.8 | Between Groups | 1314.412 | 4 | 328.603 | 40.199 | .000 |
|  | Within Groups | 196.184 | 24 | 8.174 |  |  |
|  | Total | 1510.596 | 28 |  |  |  |
| ULCER.RATE.12 | Between Groups | 508.014 | 4 | 127.004 | 10.318 | .000 |
|  | Within Groups | 270.804 | 22 | 12.309 |  |  |
|  | Total | 778.818 | 26 |  |  |  |
| ULCER.RATE.16 | Between Groups | 107.825 | 4 | 26.956 | 3.715 | .020 |
|  | Within Groups | 145.124 | 20 | 7.256 |  |  |
|  | Total | 252.949 | 24 |  |  |  |

**Post Hoc Tests**

| Dependent Variable | (I) Group | (J) Group | Sig. |
| --- | --- | --- | --- |
|  |  |  |  |
| ULCER.RATE.4 | CONTROL | CONTROL(ADS) | .030 |
|  |  | PBM+ADS(Invivo) | .000 |
|  |  | PBM+ADS(Invitro) | .033 |
|  |  | PBM+ADS(Invivo&Invitro) | .010 |
|  | CONTROL(ADS) | CONTROL | .030 |
|  |  | PBM+ADS(Invivo) | .016 |
|  |  | PBM+ADS(Invitro) | .877 |
|  |  | PBM+ADS(Invivo&Invitro) | .549 |
|  | PBM+ADS(Invivo) | CONTROL | .000 |
|  |  | CONTROL(ADS) | .016 |
|  |  | PBM+ADS(Invitro) | .033 |
|  |  | PBM+ADS(Invivo&Invitro) | .062 |
|  | PBM+ADS(Invitro) | CONTROL | .033 |
|  |  | CONTROL(ADS) | .877 |
|  |  | PBM+ADS(Invivo) | .033 |
|  |  | PBM+ADS(Invivo&Invitro) | .688 |
|  | PBM+ADS(Invivo&Invitro) | CONTROL | .010 |
|  |  | CONTROL(ADS) | .549 |
|  |  | PBM+ADS(Invivo) | .062 |
|  |  | PBM+ADS(Invitro) | .688 |
| ULCER.RATE.8 | CONTROL | CONTROL(ADS) | .000 |
|  |  | PBM+ADS(Invivo) | .000 |
|  |  | PBM+ADS(Invitro) | .000 |
|  |  | PBM+ADS(Invivo&Invitro) | .000 |
|  | CONTROL(ADS) | CONTROL | .000 |
|  |  | PBM+ADS(Invivo) | .008 |
|  |  | PBM+ADS(Invitro) | .020 |
|  |  | PBM+ADS(Invivo&Invitro) | .000 |
|  | PBM+ADS(Invivo) | CONTROL | .000 |
|  |  | CONTROL(ADS) | .008 |
|  |  | PBM+ADS(Invitro) | .700 |
|  |  | PBM+ADS(Invivo&Invitro) | .002 |
|  | PBM+ADS(Invitro) | CONTROL | .000 |
|  |  | CONTROL(ADS) | .020 |
|  |  | PBM+ADS(Invivo) | .700 |
|  |  | PBM+ADS(Invivo&Invitro) | .001 |
|  | PBM+ADS(Invivo&Invitro) | CONTROL | .000 |
|  |  | CONTROL(ADS) | .000 |
|  |  | PBM+ADS(Invivo) | .002 |
|  |  | PBM+ADS(Invitro) | .001 |
| ULCER.RATE.12 | CONTROL | CONTROL(ADS) | .015 |
|  |  | PBM+ADS(Invivo) | .000 |
|  |  | PBM+ADS(Invitro) | .000 |
|  |  | PBM+ADS(Invivo&Invitro) | .000 |
|  | CONTROL(ADS) | CONTROL | .015 |
|  |  | PBM+ADS(Invivo) | .008 |
|  |  | PBM+ADS(Invitro) | .031 |
|  |  | PBM+ADS(Invivo&Invitro) | .003 |
|  | PBM+ADS(Invivo) | CONTROL | .000 |
|  |  | CONTROL(ADS) | .008 |
|  |  | PBM+ADS(Invitro) | .566 |
|  |  | PBM+ADS(Invivo&Invitro) | .778 |
|  | PBM+ADS(Invitro) | CONTROL | .000 |
|  |  | CONTROL(ADS) | .031 |
|  |  | PBM+ADS(Invivo) | .566 |
|  |  | PBM+ADS(Invivo&Invitro) | .380 |
|  | PBM+ADS(Invivo&Invitro) | CONTROL | .000 |
|  |  | CONTROL(ADS) | .003 |
|  |  | PBM+ADS(Invivo) | .778 |
|  |  | PBM+ADS(Invitro) | .380 |
| ULCER.RATE.16 | CONTROL | CONTROL(ADS) | .154 |
|  |  | PBM+ADS(Invivo) | .035 |
|  |  | PBM+ADS(Invitro) | .024 |
|  |  | PBM+ADS(Invivo&Invitro) | .001 |
|  | CONTROL(ADS) | CONTROL | .154 |
|  |  | PBM+ADS(Invivo) | .422 |
|  |  | PBM+ADS(Invitro) | .316 |
|  |  | PBM+ADS(Invivo&Invitro) | .033 |
|  | PBM+ADS(Invivo) | CONTROL | .035 |
|  |  | CONTROL(ADS) | .422 |
|  |  | PBM+ADS(Invitro) | .837 |
|  |  | PBM+ADS(Invivo&Invitro) | .165 |
|  | PBM+ADS(Invitro) | CONTROL | .024 |
|  |  | CONTROL(ADS) | .316 |
|  |  | PBM+ADS(Invivo) | .837 |
|  |  | PBM+ADS(Invivo&Invitro) | .235 |
|  | PBM+ADS(Invivo&Invitro) | CONTROL | .001 |
|  |  | CONTROL(ADS) | .033 |
|  |  | PBM+ADS(Invivo) | .165 |
|  |  | PBM+ADS(Invitro) | .235 |
